# Identification of disease-linked hyperactivating mutations in *UBE3A* through large-scale functional variant analysis

**DOI:** 10.1038/s41467-021-27156-0

**Published:** 2021-11-23

**Authors:** Kellan P. Weston, Xiaoyi Gao, Jinghan Zhao, Kwang-Soo Kim, Susan E. Maloney, Jill Gotoff, Sumit Parikh, Yen-Chen Leu, Kuen-Phon Wu, Marwan Shinawi, Joshua P. Steimel, Joseph S. Harrison, Jason J. Yi

**Affiliations:** 1 Department of Neuroscience, Washington University School of Medicine, St. Louis, MO 63110 USA; 2 Department of Psychiatry, Washington University School of Medicine, St. Louis, MO 63110 USA; 3 Department of Pediatrics, Geisinger Medical Center, Danville, PA 17822 USA; 4 Department of Neurogenetics, Neurosciences Institute, Cleveland Clinic, Cleveland, OH 44106 USA; 5 Institute of Biological Chemistry, Academia Sinica, Taipei, Taiwan; 6 Division of Genetics and Genomic Medicine, Department of Pediatrics, St. Louis Children’s Hospital, Washington University School of Medicine, St. Louis, MO 63110 USA; 7 Deparment of Mechanical Engineering, University of the Pacific, Stockton, CA 95211 USA; 8 Department of Chemistry, University of the Pacific, Stockton, CA 95211 USA

**Keywords:** Proteolysis, Neurodevelopmental disorders, Development of the nervous system, Molecular neuroscience

## Abstract

The mechanisms that underlie the extensive phenotypic diversity in genetic disorders are poorly understood. Here, we develop a large-scale assay to characterize the functional valence (gain or loss-of-function) of missense variants identified in *UBE3A*, the gene whose loss-of-function causes the neurodevelopmental disorder Angelman syndrome. We identify numerous gain-of-function variants including a hyperactivating Q588E mutation that strikingly increases UBE3A activity above wild-type UBE3A levels. Mice carrying the Q588E mutation exhibit aberrant early-life motor and communication deficits, and individuals possessing hyperactivating *UBE3A* variants exhibit affected phenotypes that are distinguishable from Angelman syndrome. Additional structure-function analysis reveals that Q588 forms a regulatory site in UBE3A that is conserved among HECT domain ubiquitin ligases and perturbed in various neurodevelopmental disorders. Together, our study indicates that excessive UBE3A activity increases the risk for neurodevelopmental pathology and suggests that functional variant analysis can help delineate mechanistic subtypes in monogenic disorders.

## Abstract (web summary)

UBE3A gene dysregulation is associated with neurodevelopmental disorders, but predicting the function of UBE3A variants remains difficult. The authors use a high-throughput assay to categorize variants by functional activity, and show that UBE3A hyperactivity increases the risk of neurodevelopmental disease.

## Introduction

The identification of genes linked to human disorders traditionally focuses on lesions that can be interpreted easily as reducing gene function. Although this method has been effective in establishing causality, a simple loss-of-function model is insufficient to account for the broad phenotypic heterogeneity observed in many neurodevelopmental disorders. This is especially important for the millions of uncharacterized coding variants identified in the human genome^1,2^, some of which represent pathogenic mutations that may bi-directionally alter the functional valence of a protein (loss or gain-of-function). However, the clinical significance for the vast majority of known variants remains undetermined^3^, representing an immensely understudied area of human genetics.

*UBE3A* encodes an E3 ubiquitin ligase that promotes the proteasomal degradation of proteins^4^. Abnormal changes in UBE3A activity are associated with various human conditions including human papilloma virus-mediated cancer^4^ and neurodevelopmental disorders. In neurons, *UBE3A* expression is epigenetically modified such that transcription of paternal *UBE3A* is silenced and only the maternally-inherited copy is expressed^5–7^. It is well established that loss of maternal *UBE3A* causes Angelman syndrome (AS), a severe form of intellectual disability characterized by epilepsy, motor deficits, dysmorphic facial features, and a unique happy demeanor^8–10^. In contrast, excessive UBE3A activity resulting from duplication of maternal chromosome 15q11-13, the region where *UBE3A* resides, is linked to an autistic disorder known as Dup15q syndrome^11^. However, there are only two known examples that specifically link UBE3A gain-of-function to neurodevelopmental disease. This includes one de novo hyperactivating missense mutation identified in a child with autism^12,13^, and a microduplication of *UBE3A* that segregates with neuropsychiatric phenotypes in one family^14^. These limited observations have raised questions about the extent to which excessive UBE3A activity contributes to neurodevelopmental pathology.

UBE3A belongs to the HECT (Homologous to E6-AP C-terminus) domain superfamily of E3 ubiquitin ligases which are implicated in an array of human conditions^15^. They all possess the eponymous HECT domain which houses the biochemical machinery necessary to accept activated ubiquitin from E2 enzyme, and transfers it to substrate proteins. In previous work, we and others found that UBE3A activates WNT reporter gene expression in HEK293T cells in a manner that is dependent on its ubiquitin ligase activity^16–19^.

Here, we used this insight to quantitatively assess the functional significance of 152 *UBE3A* variants identified in individuals. We discovered many mutations that impact UBE3A activity, including 18 hyperactivating mutations. By generating a knock-in mouse model, we show that hyperactivation of maternal, but not paternal UBE3A is sufficient to perturb neonatal motor behaviors and communication, and using patient data, we show that UBE3A hyperactivity produces phenotypes that are distinct from AS. Finally, using the rich structure–function data derived from our screen, we uncovered a conserved regulatory mechanism in UBE3A that is commonly perturbed by both loss and gain-of-function mutations. Together, our results provide strong evidence that excessive UBE3A activity increases the risk for neurodevelopmental pathology and suggest that detailed functional variant analysis can provide a widely-applicable method to identify mechanistic sub-classes of genetic disorders.

## Results

### An efficient cell-based screen to characterize UBE3A variant function

**Fig. 1:**
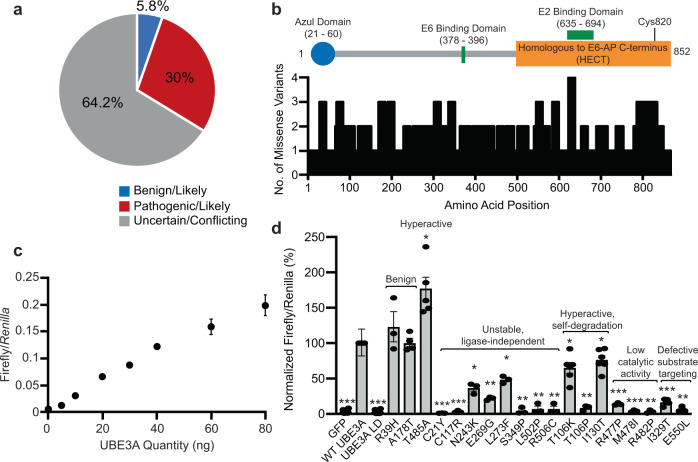
Genetic variation in UBE3A. **a** The distribution of clinical interpretations in ClinVar for missense variants identified in UBE3A. Blue: Benign/Likely Benign, Red: Pathogenic/Likely Pathogenic, Gray: Uncertain/Conflicting. **b** Schematic showing human UBE3A with known functional domains. The number of variants and their positions corresponding to the UBE3A protein sequence are shown. **c** Cultured cells were transfected with increasing amounts of plasmid encoding WT UBE3A. Mean values are shown for Firefly/*Renilla* ratios ± standard error (SE). *N* = 3 independent experiments. **d** BAR responses of variants were normalized to WT UBE3A responses in each experiment. Values are shown as the mean ± SE. Each variant was tested in triplicate per experiment and the resulting values averaged. The number of experiments performed (*n*) and *p*-values calculated using a one-sample *t*-test (two-tailed) with Benjamini–Hochberg multiple comparisons correction (FDR = 0.05) are as follows: GFP, *n* = 19, ***p = 1.733 × 10^−31^; WT UBE3A, *n* = 19; UBE3A LD, ***p = 1.519 × 10^−31^, *n* = 19; R39H, *n* = 3, *p* = 0.567; A178T, *n* = 4, *p* = 0.828; T485A, *n* = 5, *p = 0.019; C21Y, *n* = 3, ***p = 8.725 × 10^−6^; C117R, *n* = 3, ***p = 3.419 × 10^−4^; N243K, *n* = 3, **p = 0.0072; E269G, *n* = 3, **p = 7.787 × 10^−4^; L273F, *n* = 3, **p = 0.0052; S349P, *n* = 3, **p = 0.0016; L502P, *n* = 3, **p = 0.0033; R506C, *n* = 3, **p = 0.0033; T106K, *n* = 6, **p = 0.0.0078; T106P, *n* = 3, **p = 0.0013; I130T, *n* = 6, *p = 0.016; R477P, *n* = 3, ***p = 4.24 × 10^−4^; M478I, *n* = 3, ***p = 3.39 × 10^−4^; R482P, *n* = 3, **p = 5.82 × 10^−4^; I329T, *n* = 5, ***p = 1.22 × 10^−5^; E550L, *n* = 3, *p = 0.0013.

There are hundreds of non-truncating *UBE3A* missense variants contained in the Genome Aggregation (gnomAD v2.1.1)^2^ and ClinVar^1^ databases. Most variants are exceedingly rare (average allele frequency <0.0001) and nearly two-thirds of variants in ClinVar have conflicting interpretations or are classified as a variant of uncertain significance (VUS; Fig. 1a). Moreover, variants in *UBE3A* occur both inside and outside of known functional domains, making it impossible to predict their functional significance without empirical assessment (Fig. 1b).

We utilized the luciferase-based β-catenin activity reporter (BAR)^20^, which is activated in a dose-dependent way by UBE3A (Fig. 1c), to determine if it can accurately assess the activity of *UBE3A* variants. We tested a variety of variants clinically and functionally validated in previous studies^12,21–29^. Among these were the benign variants R39H and A178T^28,29^, the autism-linked hyperactivating mutation T485A^12,13^, as well as numerous AS-linked mutations known to cause loss-of-function through various mechanisms^12,21^. We introduced mutations into a plasmid encoding human UBE3A isoform 2 and measured the BAR response in HEK293T cells. For every mutant, the BAR assay correctly identified changes in the valence and severity of the mutation in relation to wild type (WT) UBE3A (Fig. 1d). Whereas the benign R39H (119.68% ± 21.3) and A178T (97.45% ± 5.9) variants did not deviate from WT UBE3A activity levels, the T485A mutant increased the BAR response (189.02% ± 12.4) and all AS-linked loss-of-function mutations exhibited diminished responses regardless of their mechanism of dysfunction. This included constitutively self-targeting mutations whose severity was demonstrated previously to be strong (T106P, 8.45% ± 1.8) and mild (T106K, 65.09% ± 5.1 and I130T, 76.31% ± 6.1), indicating that BAR responses can distinguish the relative severity of UBE3A dysfunction^12^. Together, these results demonstrate the utility of the BAR assay as a general and accurate reporter of UBE3A activity across different mechanisms that perturb enzyme function.

### UBE3A variants define a broad landscape of functional effects

**Fig. 2:**
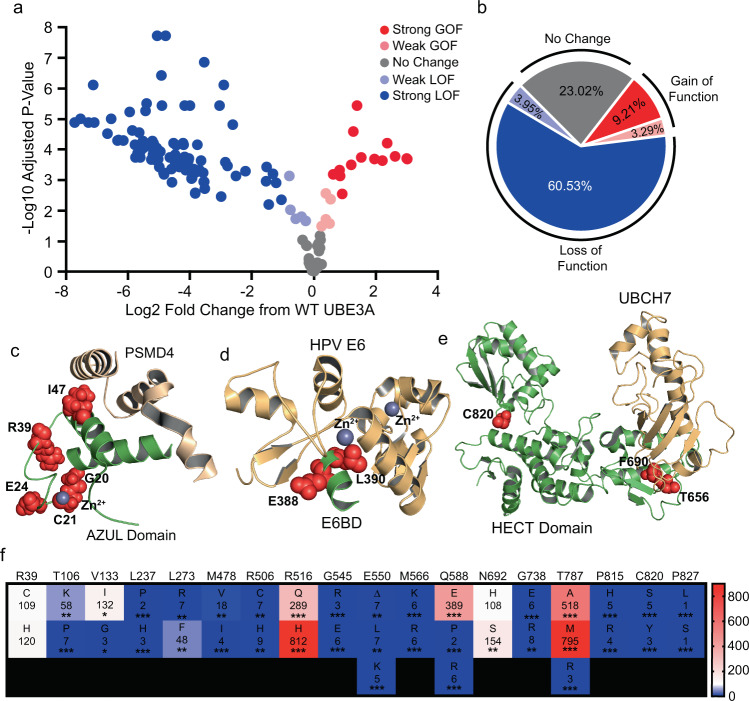
UBE3A variants encompass a broad landscape of functional effects. **a** BAR assay screen of 152 UBE3A variants showing benign (gray), weak loss-of-function (light blue), strong loss-of-function (blue), weak gain-of-function (pink), and strong gain-of-function (red) mutations relative to WT UBE3A. Significance was determined using a One-sample *t*-test (two-tailed) with Benjamini–Hochberg multiple comparisons correction (FDR = 0.05). Exact numbers of experiments and *p*-values are provided in Supplementary Data 1. Red, strong gain-of-function; Pink, weak gain-of-function; Gray, no change from WT UBE3A; Light blue, weak loss-of-function; Blue, strong loss-of-function. **b** Distribution of functional classes for variants tested in our screen. **c** Solution NMR (PDB: 6U19) of the AZUL domain of UBE3A (green) bound to PSMD4 (gold). Variants tested in our screen are shown in red. **d** Co-crystal structure (PDB: 4GIZ) of the E6BD domain of UBE3A (green) bound to HPV E6 (gold). Variants tested in our screen are shown in red. **e** Co-crystal structure (PDB: 1C4Z) of the HECT domain of UBE3A (green) bound to the E2 enzyme UBCH7 (gold). Variants tested in our screen (red) included mutations at the catalytic cysteine (C820) and the E2-binding interface (T656 and F690). **f** Heat map plot showing BAR assay results from UBE3A sites with multiple variants. Values are normalized to WT UBE3A activity. White shading represents WT UBE3A activity levels, blue shading indicates loss-of-function, and red shading indicates gain-of-function. Scale bar shows the percent change relative to WT UBE3A. Exact numbers of experiments and *p*-values are provided in Supplementary Data 1, *p < 0.05, **p < 0.005, *p* < 0.0005, One-sample *t*-test (two-tailed) with Benjamini–Hochberg multiple comparisons correction (FDR = 0.05).

We cloned nearly all of the *UBE3A* missense variants present in the ClinVar database and assessed their effect on UBE3A activity (Fig. 2a). Our library contained 152 variants in total, comprised of 133 amino acid substitutions, 18 in-frame deletions, and one in-frame duplication. Whereas the activity of ~23% of variants did not change from WT UBE3A, indicating they are likely benign, we classified ~61% as strong loss-of-function mutations (≤50% of WT UBE3A activity) and ~4% as weak loss-of-function mutations (>50% of WT UBE3A activity; Fig. 2b and Supplementary Data 1). Surprisingly, our screen also found that ~9% of all variants are strong gain-of-function mutations (>150% of WT UBE3A activity) while ~3% are weak gain-of-function mutations (≤150% of WT UBE3A activity; Fig. 2b and Supplementary Data 1).

Some of the variants tested in our screen disrupted residues in domains known to be important for UBE3A function. One such domain was an N-terminal zinc (Zn^2+^) finger known as the AZUL domain (amino-terminal Zn-finger of UBE3A ligase) that dictates UBE3A localization^19,30,31^. Six variants were located within the AZUL domain including a loss-of-function mutation that disrupts a Zn^2+^ chelating residue (C21Y, 1.07% ± 0.001 of WT UBE3A activity) and the adjacent amino acid (G20V, 2.07% ± 0.79; Fig. 2c). Our screen also included variants in the E6 binding domain (E6BD; amino acids 378–396), a site where the human papilloma virus (HPV) E6 protein binds to UBE3A^4^. Two hyperactivating mutations were found within this region including a L390F mutation that elevated UBE3A activity 229.81% ± 9.33 above the WT BAR response (Fig. 2d), suggesting the E6BD is an important regulator of UBE3A function even in the absence of HPV E6. Finally, we identified a large number of mutations in the HECT domain of UBE3A, including loss-of-function mutations in the E2 enzyme binding domain (T656I and F690C)^32^, and loss-of-function mutations at the catalytic cysteine (C820S and C820Y; Fig. 2e)^33^.

Our functional data correlated poorly to predictions for two in silico programs used widely for variant interpretation^34,35^ (Supplementary Fig. 1a), demonstrating the necessity for empirical assessment. A particularly interesting class of variants were substitutions that occurred at the same amino acid position. There were 18 such cases comprising a total of 39 variants tested in our study (Fig. 2f). Some of these variants had consistent effects and could be classified collectively as benign (R39C/H), loss-of-function (T106K/P, L237H/P, L273R/F, M478V/I, R506C/H, G545R/E, E550Δ/L/K, M566K/R, G738E/R, P815H/R, C820S/Y, and P827L/S), or gain-of-function (R516Q/W). In contrast, asparagine 692 (N692) possessed a benign (N692H) and a weak gain-of-function mutation (N692S) whereas valine 133 (V133) possessed a weak gain-of-function (V133I) and strong loss-of-function mutation (V133G). Moreover, glutamine 588 (Q588) and threonine 787 (T787) simultaneously possessed strong gain-of-function mutations (Q588E and T787A/M) and strong loss-of-function (Q588P/R and T787R) mutations (Fig. 2f).

We were particularly interested in this last class of mutations because they exerted a disproportionally large and bi-directional influence on UBE3A activity, suggesting that sites containing hyperactivating mutations in general, are hyper-modulatory sites that mediate the active and inactive states of UBE3A. To test this hypothesis, we substituted every possible amino acid at leucine 781 (L781) and T787, two positions that contained the strongest hyperactivating mutations in our screen (L781H and T787M; Supplementary Fig. 1b). We anticipated that mutation of these sites would produce multiple strong loss and gain-of-function enzymes reflecting their central role in enzyme regulation. Indeed, we found an extensive range of effects in our BAR assay that spanned from 7.48 to 656.55% of WT UBE3A activity for L781, and 1.33 to 1245.07% of WT UBE3A activity for T787 (Supplementary Fig. 1b and Supplementary Table 1). Taken together, our analyses demonstrate that variants in *UBE3A* impart highly heterogeneous effects to protein function and suggest that sites of hyperactivating mutations signify amino acids that are critical for UBE3A regulation.

### Hyperactivating mutations alter UBE3A ubiquitin ligase activity

**Fig. 3:**
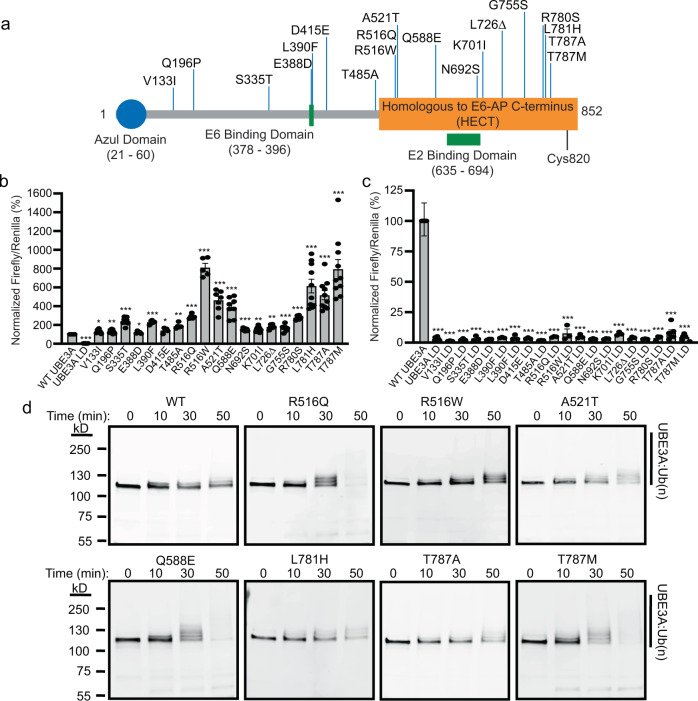
Characterization of hyperactivating mutations in UBE3A. **a** Schematic of UBE3A showing the positions of hyperactivating mutations identified in our screen. **b**, **c** Normalized BAR responses of hyperactivating mutations with (**b**) and without (**c**) a ligase-dead (LD; C820A) mutation. BAR responses in **b** were re-plotted from the initial screen in Fig. 2 and are shown as the mean ± SE. Exact numbers of experiments and *p*-values for variants with ligase activity are provided in Supplementary Data 1. All variants for the LD were tested in three independent experiments and *p*-values calculated using a One-sample *t*-test (two-tailed) with Benjamini–Hochberg multiple comparisons correction (FDR = 0.05). V133I LD, ***p = 8.68 × 10^−6^; Q196P LD, ***p = 3.56 × 10^−5^; S33T LD, ***p = 1.49 × 10^−4^; E388D, ***p = 1.44 × 10^−4^; L390F LD, ***p = 1.20 × 10^−4^; D415E LD, ***p = 1.68 × 10^−4^; T485A LD, ***p = 2.36 × 10^−5^; R516Q LD, ***p = 1.55 × 10^−5^; R516W LD, ***p = 1.59 × 10^−4^; A521T LD, **p = 0.0014; Q588E LD, ***p = 1.25 × 10^−4^; N692S, ***p = 1.37 × 10^−5^; K701I, ***p = 1.37 × 10^−5^; L726Δ LD, ***p = 1.02 × 10^−4^; G755S LD, ***p = 6.25 × 10^−5^; R780S LD, ***p = 8.68 × 10^−6^; L781H LD, ***p = 1.44 × 10^−4^; T787A LD, ***p = 1.36 × 10^−6^; T787M LD, ***p = 1.25 × 10^−8^. **d** In vitro ubiquitination assay was performed using UBE3A mutants expressed and purified from bacteria. Reactions were stopped at the indicated times and the formation of self-ubiquitinated UBE3A was monitored by western blot using an anti-UBE3A antibody. Representative images are shown from three independent experiments that produced similar results.

To date, the UBE3A T485A mutation, which abolishes a regulatory PKA phosphorylation site, is the only disease-linked gain-of-function mutation reported for UBE3A^12^. Our analysis identified 18 additional hyperactivating mutations (Fig. 3a) with many exhibiting much higher activity than UBE3A T485A (Fig. 3b). We sought to validate whether these variants represented a true gain-of-function of the ubiquitin ligase activity of UBE3A. Western blot analysis showed that all gain-of-function mutants were present at comparable abundance or were less abundant than WT UBE3A in HEK293T cells (Supplementary Fig. 2a, b), indicating hyperactivity did not result from a simple increase in enzyme levels. Moreover, when we introduced a ligase-dead mutation (C820A) into each gain-of-function mutant, there was a complete loss of the BAR response (Fig. 3c), demonstrating that reporter activation resulted from increased ubiquitin ligase activity.

We next tested the ability of gain-of-function mutants to self-ubiquitinate, a process shown to be accelerated for the hyperactive UBE3A T485A mutation^12^ as well as for a variety of active HECT domain ubiquitin ligases^36,37^. We selected several strong gain-of-function mutations in the HECT domain and performed in vitro ubiquitination reactions using recombinant proteins purified from bacterial cells. We found that mutant proteins accelerated self-ubiquitination resulting in the formation of higher molecular weight bands and the eventual loss of the monomeric UBE3A band (Fig. 3d). This result was consistent in HEK293T cells where gain-of-function mutants increased self-ubiquitination compared to WT UBE3A (Supplementary Fig. 2c). Finally, we measured the ability of gain-of-function mutants to target a substrate protein for degradation in cells. We performed this experiment with a ligase-inactive mutant of RING1B, a polycomb repressor complex protein used widely as an in vitro substrate of UBE3A^38^. Western blot analysis showed that whereas RING1B abundance increased in the presence of ligase-dead UBE3A (176% ± 0.13 relative to WT), every hyperactivating mutation tested in our assay decreased RING1B protein levels. Indeed, R516Q (67.48% ± 3.68), R516W (60.16% ± 4.63), A521T (55.16% ± 4.71), Q588E (60.58% ± 4.44), L781H (52.31% ± 4.30), T787A (52.96% ± 6.31), and T787M (52.37% ± 4.91) all reduced RING1B abundance to a fraction of WT UBE3A levels (Supplementary Fig. 2d, e). Collectively, these results provide direct evidence that gain-of-function mutations identified in our study elevate the ubiquitin ligase activity of UBE3A.

### Structure–function analysis reveals a degenerate exosite in UBE3A

**Fig. 4:**
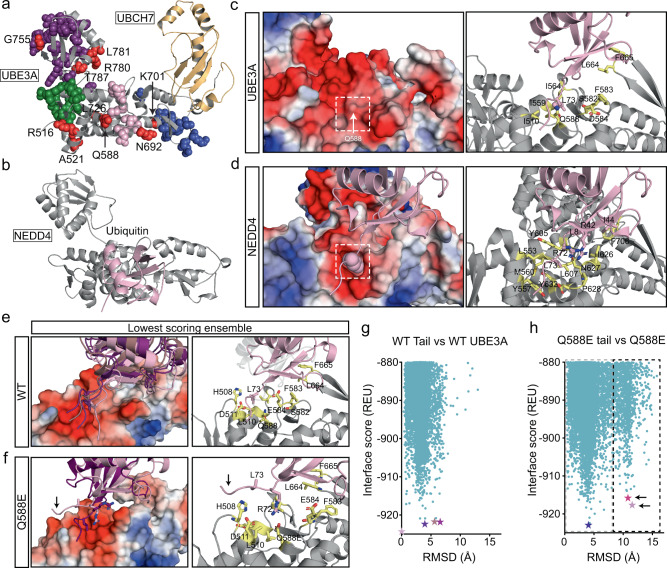
UBE3A mutations demarcate a ubiquitin-binding exosite. **a** Crystal structure (PDB: 1C4Z) of the HECT domain of UBE3A (gray) bound to UBCH7 (gold). Mutations identified in our screen found in the catalytic C-lobe (purple), the E2 binding interface (blue), and two uncharacterized regions (pink and green) are shown. All hyperactivating mutations are shown in red. **b** Crystal structure of the HECT domain of NEDD4 (gray) bound to a monomeric ubiquitin molecule (pink) at its exosite (PDB: 4BBN). Note the binding of ubiquitin to NEDD4 occurs at a site homologous to a cluster of mutations (pink) in UBE3A. **c**, **d** Surface (left) and ribbon (right) representations of the ubiquitin-binding interface for UBE3A (**c**) and NEDD4 (**d**). Ubiquitin is represented in pink. Dotted boxes represent the position of hydrophobic binding pocket in NEDD4 that accepts L73 of the ubiquitin C-terminus. Surface charges are indicated by color: positive (blue), negative (red), uncharged (white). **e**, **f** Rosetta simulations showing ensembles of low energy (high confidence) conformations for the C-terminus of ubiquitin docked to UBE3A. The divergent orientation of the ubiquitin tail caused by the Q588E mutation in UBE3A is noted by the arrow. **g**, **h** Interface score versus RMSD plots showing Rosetta simulations under 880 Rosetta Energy Units (REU). The positions of the low energy models are noted as stars according to the color scheme used in **e**, **f**. The gray dotted box demarcates WT-like tail conformations whereas the black dotted box indicates the Q588E tail conformation.

The rich functional information provided by our screen allowed us to perform extensive structure-function analyses of UBE3A. When we mapped the spatial distribution of all loss and gain-of-function mutations identified in our screen, we found they localized to several structural features in the UBE3A HECT domain (Fig. 4a). This included the C-lobe, which contains the catalytic cysteine required for ubiquitin transfer^25^, a number of mutations in the E2 binding region^32^, a group in a region of unknown function, and one group that demarcated a recently characterized non-covalent ubiquitin binding site in UBE3A known as the exosite^39^. The exosite is best characterized in NEDD4 subfamily HECT domain enzymes, where studies showed that binding of monomeric or dimeric ubiquitin mediates ubiquitin ligase activity^36,40–43^. Previous structural and mutational analyses determined that ubiquitin binding at the exosite requires two principal contact points in NEDD4^42^: a hydrophobic patch formed by phenylalanine 707 (F707) in NEDD4 and isoleucine 44 (I44) in ubiquitin, and another hydrophobic contact formed by two tyrosines (Y605 and Y634) that interact with leucine 73 (L73) near the C-terminus of ubiquitin (Fig. 4b and Supplementary Fig. 3a). Sequence alignment of the ubiquitin contacting domain in UBE3A showed a high degree of conservation across species (Supplementary Fig. 3b). However, whereas the NEDD4 F707 position is conserved in UBE3A (F665 in UBE3A), UBE3A lacks the aromatic residues at Y605 and Y634 (I564 and Q588, respectively) that are present in every NEDD4 subfamily enzyme (Supplementary Fig. 3c).

Our observations raised the possibility that UBE3A possesses a degenerate exosite that binds ubiquitin through mechanisms that diverge from NEDD4 subfamily enzymes. To explore this possibility, we used the crystal structure of the UBE3A HECT domain and the co-crystal structure of ubiquitin bound to the NEDD4 exosite (Fig. 4b; PDB: 4BBN) to perform constrained rigid-body docking with the Rosetta molecular modeling program^44^. Modeling showed that F665 in UBE3A contacts I44 of ubiquitin, and in functional experiments, we found that mutation of F665 to alanine (F665A) caused a near-complete loss of UBE3A activity in our BAR assay (Supplementary Fig. 3d), demonstrating that F665 is indispensable for UBE3A activity. In contrast, there were large structural changes at the second ubiquitin binding site. Y605 and Y634 in NEDD4 contribute to form a deep hydrophobic pocket that permits a stable interaction with the side chain of L73 in ubiquitin (Fig. 4d). However, this pocket is shallow in UBE3A and superimposing the orientation of ubiquitin bound to NEDD4 resulted in steric clashing between atoms in the sidechains of Q588 in UBE3A and L73 in ubiquitin (Fig. 4c).

### Position Q588 is critical for ubiquitin binding at the UBE3A exosite

Intriguingly, there were three mutations at the Q588 position tested in our screen including a substitution to glutamate (Q588E), proline (Q588P), and arginine (Q588R), all of which altered the overall charge in the ubiquitin-binding pocket of UBE3A (Supplementary Fig. 3e). The Q588E mutation was a gain-of-function mutation that elevated UBE3A activity 389% ± 35.8 above WT UBE3A levels (Fig. 3b and Supplementary Data 1) whereas Q588P and Q588R abolished UBE3A ubiquitin ligase activity (Supplementary Data 1). These functional observations suggested that Q588 is a critical residue that mediates exosite function in UBE3A.

To further interrogate how Q588 impacts ubiquitin binding, we used the Relax application in the Rosetta Macromolecular modeling suite^45^, starting from the orientation of ubiquitin in the NEDD4:ubiquitin co-crystal structure. High confidence models placed ubiquitin in a similar conformation relative to its starting position, and key contacts typically made by ubiquitin when bound to NEDD4 subfamily exosites were preserved in UBE3A. Further analysis revealed an ensemble of closely-aligned conformations in which the C-terminus of ubiquitin, including L73, bound UBE3A through a series of interactions surrounding the shallow hydrophobic pocket, including contacts with Q588 (Fig. 4e). Root mean square deviation (RMSD) versus Rosetta score plots showed a broad funnel that corresponds to this ensemble of similar C-terminal tail conformations (Fig. 4g). We repeated this modeling with the Q588E variant and found that RMSD versus Rosetta score plots revealed two divergent populations of high-confidence structures that corresponded to distinct ubiquitin tail conformations docked to the Q588E exosite (Fig. 4f, h). The first population was similar to ubiquitin bound to WT UBE3A (Fig. 4f). In contrast, the second set had an alternative ubiquitin tail conformation in which the positive charge from the adjacent arginine 72 (R72) residue in ubiquitin contacted the negative charge resulting from the Q588E mutation in UBE3A. Reanalysis of the WT modeling with this alternative tail revealed there were four structures out of ~30,000 that had this similar tail. Together, our analysis indicated that Q588E mutation alters the mechanism by which ubiquitin binds to UBE3A.

### The Q588E mutation enhances ubiquitin binding to the exosite

**Fig. 5:**
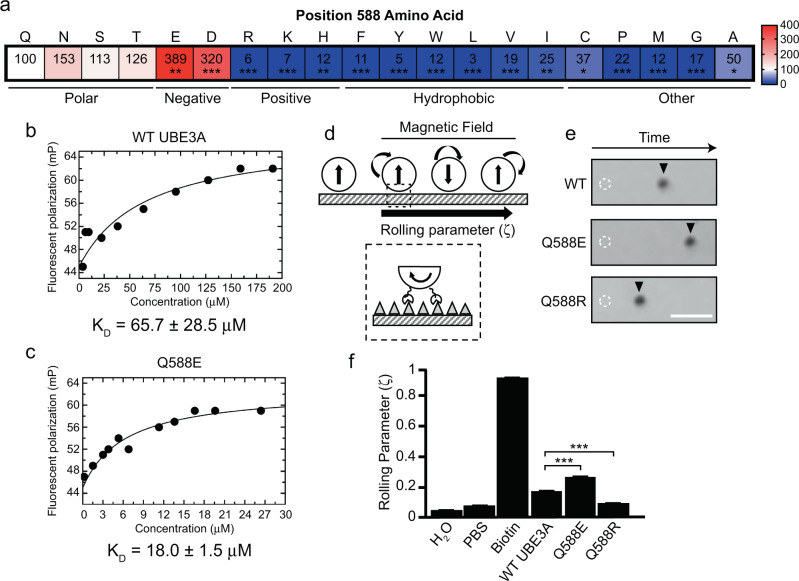
The charge at Q588 dictates ubiquitin binding and UBE3A activity. **a** Heat plot showing normalized BAR values of mutations at position 588 in UBE3A. White shading represents WT UBE3A activity levels, blue shading indicates loss-of-function, and red shading indicates gain-of-function. Scale bar shows the percent change relative to WT UBE3A. *N* = 3 independent experiments for Q588S, Q588T, Q588N, Q588K, Q588P, Q588M, Q588G, Q588A, Q588F, Q588Y, Q588W, Q588L, Q588V, Q588I, Q588C; *n* = 8 for Q588E, *n* = 6 for Q588D, *n* = 5 for Q588R, *n* = 4 for Q588H, *p < 0.05, **p < 0.005, ***p < 0.0005, One-sample *t*-test (two-tailed) with Benjamini–Hochberg multiple comparison correction (FDR = 0.05). **b**, **c** Fluorescence polarimetry to obtain dissociation constants (*K*_D_) for ubiquitin binding to UBE3A. **d**, **e** Schematic (**d**) and representative images (**e**) from rolling magnetic probe assays with beads conjugated to ubiquitin rolling on coverslips coated with WT, Q588E, and Q588R HECT domains from UBE3A. Dashed circle represents bead position at *t* = 0 and arrowhead represents bead position *t* = 5 s after a magnetic field was applied. Scale bar = 25 μm. **f** Plot showing mean ± SE of rolling parameters (ζ) of different conditions. H_2_O and phosphate buffered saline (PBS) were used as negative controls and biotinylated beads rolling on streptavidin-coated coverslips were used as a positive control. Note the ζ of Q588R is near negative control values. WT, *n* = 58; Q588E, *n* = 71; Q588R, *n* = 42, ***p < 0.0001, One-way ANOVA with Tukey’s multiple comparisons test.

Previous studies showed that ubiquitin binding to the exosite of HECT domain enzymes facilitates its ubiquitin ligase activity^36,39,41–43,46^. We performed a series of experiments to explore if the charge at amino acid position 588 alters the affinity of ubiquitin binding to UBE3A. We began by performing comprehensive mutation analysis at position Q588 in UBE3A (Fig. 5a). We tested a series of mutations at the Q588 site and assessed UBE3A activity using the BAR assay (Fig. 5a and Supplementary Table 1). Consistent with a charge-dependent interaction between Q588E of UBE3A and R72 of ubiquitin, mutation to a negatively-charged aspartate (Q588D) increased UBE3A activity 320.25% ± 30 above WT UBE3A, whereas mutation to a positively-charged lysine (Q588K, 6.60% ± 2.81) or histidine (Q588H, 12.20% ± 1.62) resulted in an inactive enzyme. Hydrophobic or uncharged residues, including mutation to aromatic residues present in NEDD4 sub-family enzymes (Q588Y and Q588W), caused UBE3A to exhibit little activity (5.10% ± 0.97 and 11.99% ± 0.62, respectively), further suggesting the mechanism of ubiquitin binding to the UBE3A exosite is divergent from NEDD4 sub-family enzymes. Finally, we found that polar residues such as serine (Q588S; 112.62% ± 11.27), threonine (Q588T; 125.72% ± 10.74), or asparagine (Q588N; 153.53% ± 11.91) all produced an enzyme with comparable activity to WT UBE3A, suggesting that Q588 participates in hydrogen bonding with ubiquitin in its native state.

We next tested whether the Q588E mutation enhances ubiquitin binding to UBE3A. We first accomplished this task using fluorescence polarimetry. Our experiments demonstrated that ubiquitin bound the WT HECT domain with a dissociation constant (*K*_D_) of 65.7 ± 28.5 μM (Fig. 5b), which was in line with a previous report^39^, but bound with higher affinity to the Q588E HECT domain with a *K*_D_ of 18.0 ± 1.5 μM (Fig. 5c). Second, we utilized a new technique termed the mechanically transduced immunosorbent (METRIS) assay^47^. In brief, this system measures weak protein interactions by utilizing a protein that is covalently conjugated to ferromagnetic particles placed on coverslips pre-coated with another protein of interest (Fig. 5d). A rotating external magnetic field is applied, which causes the beads to spin in place. If there is an interaction between the two proteins of interest, the bead migrates along the coverslip (Fig. 5e), and the extent of this linear displacement is dependent on the strength of the protein–protein interaction in the system. This displacement is captured as the rolling parameter (ζ), and the value of the rolling parameter scales in proportion with the affinity of the interaction (see “Methods” section for detailed information). We performed this experiment by conjugating ubiquitin to ferromagnetic particles and calculating its rolling parameter on UBE3A HECT domain-coated coverslips. We observed that WT UBE3A yielded a rolling parameter of 0.178 ± 0.0056, whereas the Q588E mutation increased the rolling parameter to 0.247 ± 0.0107, indicating enhanced affinity between the two proteins, and the Q588R mutation reduced the rolling parameter to 0.114 ± 0.0021 (Fig. 5f). These results confirmed our fluorescence polarimetry results that Q588E mutation enhances ubiquitin binding.

Our findings provide the first characterization of disease-relevant mutations in the exosite of any HECT domain enzyme. When we examined reported variants for other HECT domain proteins, we found additional variants classified as VUS and likely pathogenic/pathogenic in various disease contexts, including several neurodevelopmental disorders (Supplementary Fig. 4). Taken together, our experiments provide strong evidence that ubiquitin binding to the UBE3A exosite is essential for proper enzyme activity, and dysfunction at the exosite is a disease-relevant mechanism that is shared across several human disorders.

### Excessive activity of maternal UBE3A is sufficient to cause neonatal behavioral impairments in mice

**Fig. 6:**
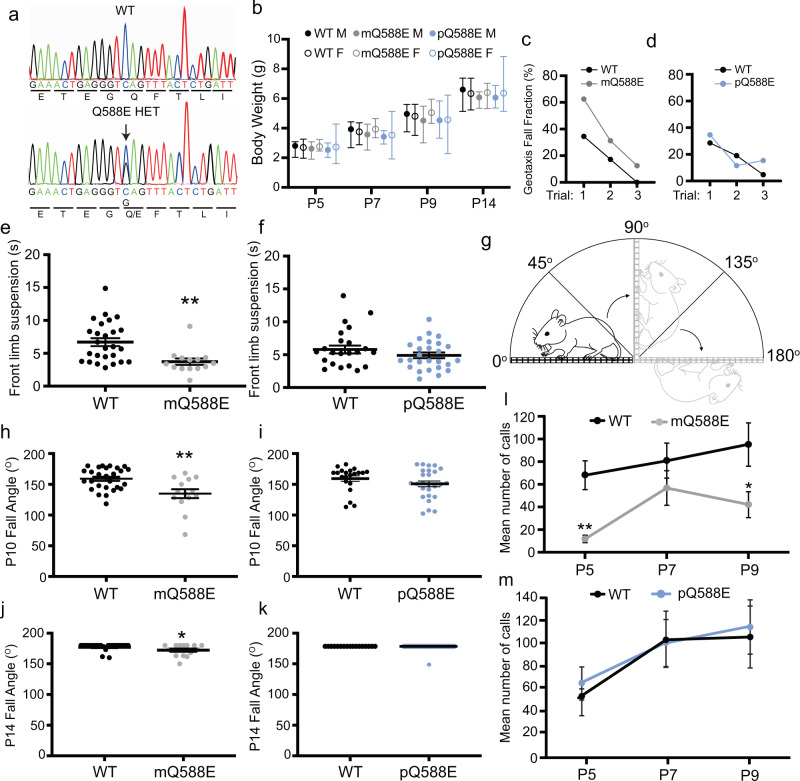
UBE3A Q588E mutation perturbs neurological function in a mouse model. **a** The genomic region containing the coding sequence for position Q588 in *UBE3A* was amplified and sequenced from WT and mQ588E mice. The arrow marks the mutated nucleotide. **b** Mean ± range of body weights of WT and mutant mice at postnatal (P) day 5, 7, 10, and 14. Solid black: WT males (WT M), Open black: WT females (WT F), Solid gray: mQ588E males (mQ588E M), Open gray: mQ588E females (mQ588E F), Solid blue: pQ588E males (pQ588E M), Open blue: pQ588E females (pQ588E F). The following numbers of animals were used for each group: WT M: P5, *n* = 13; P7, *n* = 13; P9, *n* = 10; P14, *n* = 11. WT F: P5, *n* = 10; P7, *n* = 10; P9, *n* = 9; P14, *n* = 6. mQ588E M: P5, *n* = 7; P7, *n* = 7; P9, *n* = 6; P14, *n* = 5. mQ588E F: P5, *n* = 9; P7, *n* = 9; P9, *n* = 8; P14, *n* = 5. pQ588E M: P5, *n* = 12; P7, *n* = 9; P9, *n* = 11; P14, *n* = 8. pQ588E F: P5, *n* = 18; P7, *n* = 16; P9, *n* = 15; P14, *n* = 16. **c**, **d** Mean values for the fraction of mQ588E (**c**, gray) and pQ588E (**d**, blue) animals that fell from the negative geotaxis apparatus over three trials are shown. Values reflect the average of three trials per animal. WT, *n* = 29 animals; mQ588E, *n* = 16 animals in **c**; WT, *n* = 21 animals; pQ588E, *n* = 26 animals in **d**. **e**, **f** Individual times and the mean ± SE for mQ588E (**e**, gray) and pQ588E (**f**, blue) mice in the front limb suspension test are shown. Each animal was tested three times and the average of the three trials was used in for analysis. WT, *n* = 29 animals; mQ588E, *n* = 16 animals in **e**; WT, *n* = 21 animals; mQ588E, *n* = 26 animals in **f**, **p = 0.001, Mann–Whitney *U*-test, exact significance (two-tailed). **g**–**k** Schematic (**g**) and individual and mean ± SE values for the four-paw grip strength test at mQ588E (**h**, **l** gray) and pQ588E (**j**, **k** blue) animals. Animals were tested at P10 (**h**, **j**) and at P14 (**I**, **k**) as indicated. **p = 0.001 (**h**), *p = 0.026 (**j**), Mann–Whitney *U*-test, exact significance (two-tailed). WT, *n* = 29 animals; mQ588E, *n* = 16 animals (**h**, **j**); WT, *n* = 21 animals; pQ588E, *n* = 26 animals (**i**); WT, *n* = 17 animals and pQ588E, *n* = 22 animals (**k**). **l**, **m** Mean ± SE values showing the number of calls at P5, P7, and P9 for WT (black) and mQ588E (gray) littermates (**l**), and in WT (black) and pQ588E (blue) littermates (**m**); **p = 0.001 at P5, *p = 0.038 at P9, Repeated measures ANOVA. WT: P5–P7, *n* = 31 animals; mQ588E: P5–P9, *n* = 23 animals (**l**); WT: P5 and P7, *n* = 21 animals, P9, *n* = 15 animals; pQ588E: P5, *n* = 32 animals, P7, *n* = 27 animals, P9, *n* = 26 animals (**m**).

We next considered the biological impact of UBE3A hyperactivating mutations and their potential to contribute to pathology. We used CRISPR-Cas9 recombination to create a line of knock-in mice possessing a Q588E mutation in *UBE3A* (Fig. 6a). All mutant animals were born at expected Mendelian ratios and possessed indistinguishable body weights compared to WT littermates (Fig. 6b), indicating the absence of overt physical defects or delays in growth.

We began our characterization using heterozygous *Ube3a*^*mQ588E/pWT*^ mice (mQ588E; Supplementary Fig. 5a), which possess the Q588E mutation on the maternally-inherited allele, and is thus expressed in neurons. Western blot analysis of the cortex in mQ588E animals showed reduced mutant UBE3A protein levels (Supplementary Fig. 5b). These observations were consistent with enhanced self-targeted ubiquitination and reduced steady-state levels observed previously for highly active ubiquitin ligases^36,37^, including UBE3A^12,48^ (also see Supplementary Fig. 2a–c). We tested early life behaviors in our animals using Fox’s battery of tests, which are designed to assess sensorimotor development in juvenile mice^49,50^. We first performed the surface righting reflex which measures the ability of pups to right themselves when placed in a prone dorsal position. In postnatal day 10 (P10) animals, we found that mQ588E pups accomplished this task just as effectively as their respective WT littermates (WT = 0.79 s ± 0.04, mQ588E = 0.88 s ± 0.09, Supplementary Fig. 5c). Moreover, we found no differences between WT and mutant animals when testing forepaw and hindpaw grasping reflexes (Supplementary Fig. 5d, e). Next, we used the negative geotaxis assay which measures how quickly an animal repositions itself vertically after being placed facing down a 45° slope. Although we found no differences between WT (56.59 s ± 6.46) and mQ588E (62.65 s ± 8.94) animals in their ability to rotate (Supplementary Fig. 5f), we observed that a much larger fraction of mQ588E mice consistently fell from the testing apparatus as compared to WT mice (Fig. 6c). This effect was specific to mQ588E mice as *Ube3a*^*mWT/pQ588E*^ animals possessing a paternally-inherited mutation (pQ588E; Supplementary Fig. 5a) were indistinguishable from WT littermates (Fig. 6d).

Our observations suggested that reflexive responses remained largely intact, but raised the possibility that mQ588E animals possessed deficits in strength and motor coordination. This led us to perform two additional tests in P10 mice. First, we performed the front-limb suspension test and found that mQ588E on average remained suspended on an elevated wire half as long as WT littermates (WT = 7.73s ± 1.34, mQ588E = 3.82s ± 0.42) whereas no effect was seen in pQ588E animals (WT = 4.90 ± 0.52, pQ588E = 4.13 ± 0.38; Fig. 6e, f). Next, we tested four-paw grip strength by measuring the ability of pups to remain on a wire mesh rotated slowly from a horizontal position (0°) to an inverted position (180°; Fig. 6g). At P10, mQ588E animals performed poorly and fell from the mesh at an average tilt angle of 129.48° ± 7.33 compared to 158.47° ± 3.34, for WT littermates (Fig. 6h). When we re-tested these animals at P14, we found that mQ588E mice drastically improved in their performance (171.56 ± 2.65) and were nearly indistinguishable from WT littermates (177.33 ± 1.31; Fig. 6j), suggesting that motor development was delayed in mQ588E mice. Meanwhile, pQ588E mice showed no significant differences from WT littermates at either timepoint (P10: WT = 157.70 ± 4.26, pQ588E = 148.81 ± 4.64; P14: WT = 180.0 ± 0, pQ588E = 178.64 ± 1.36; Fig. 6i, k).

Finally, we measured ultrasonic vocalizations (USVs) to determine whether early-life communication is affected in mQ588E animals. We found that on average, the number of USVs produced by WT animals increased gradually from 68.11 ± 12.67 calls at P5, to 80.81 ± 15.53 at P7, and 95.03 ± 19.10 at P9. In contrast, mQ588E animals produced dramatically fewer calls at P5 (12.57 ± 3.32) and at P9 (42.04 ± 11.55; Fig. 6l). Intriguingly, these results were opposite to neonatal Angelman syndrome model mice, which were shown to produce increased vocalizations^51^. We also observed higher pitched vocalizations, shorter call durations, and longer pause durations (Supplementary Fig. 5g–i) to varying degrees in mQ588E animals. In contrast, there were no differences in the number of vocalizations at any timepoint between pQ588E mice and their WT littermates (Fig. 6m), and a very subtle difference in pitch frequency at P5 (Supplementary Fig. 5j) with no differences in call duration or pause duration (Supplementary Fig. 5k, l). Taken together, our observations provide direct evidence that hyperactivation of maternal, but not paternal UBE3A, is sufficient to delay motor development and produce deficits in early life communication.

### Hyperactivating mutations in UBE3A may contribute to neurodevelopmental phenotypes that are distinct from Angelman syndrome

**Fig. 7:**
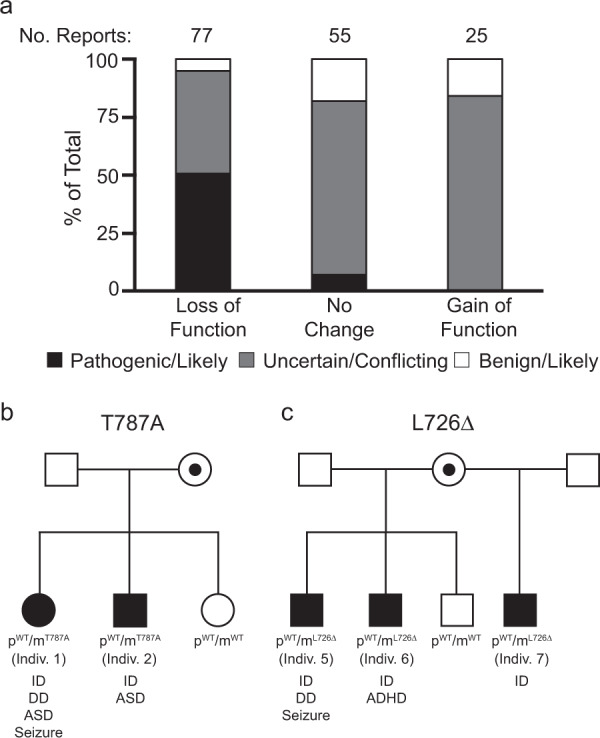
UBE3A hyperactivity causes neurodevelopmental dysfunction. **a** Clinical interpretations in ClinVar for variants characterized in this study. The number of individual reports contained in ClinVar are shown for pathogenic/likely pathogenic (black), uncertain/conflicting (gray), and benign/likely benign (white) variants. **b**, **c** Pedigree chart for the T787A (**b**) and L726Δ (**c**) hyperactive mutations. ID intellectual disability, DD developmental delay, ASD autism spectrum disorder, ADHD attention-deficit/hyperactivity disorder.

Although AS is clinically recognizable^52^, phenotypes caused by excessive UBE3A activity are not well documented. Thus, when we compared our functional screening results with variant classifications in the ClinVar database, we predicted that a greater number of loss-of-function variants would be classified as pathogenic whereas gain-of-function mutations would be classified as VUS. Consistent with our hypothesis, our data showed that about half (50.6%) of loss-of-function mutations identified in our screen were deemed pathogenic whereas most gain-of-function mutations were classified as VUS (84%) with a few designated as benign (16%) and none designated as pathogenic (Fig. 7a).

To gain further clarity into human phenotypes, we collaborated with clinical centers to collect data from 17 individuals possessing hyperactivating mutations identified in our study. A summary of their clinical phenotypes is presented in Supplementary Table 2 and a detailed account for each individual is presented in our Supplementary Information (Supplementary Note 1). Our data indicated that hyperactivating mutations are typically maternally inherited, and individuals generally exhibit intellectual disability, seizures, and autism. In some cases, phenotypes distinct from either classic AS or Dup15q were reported. However, none of the individuals examined in our study were diagnosed with AS.

Here we highlight two families in which mutations segregated with affected individuals. Individual 1 was an adult who possessed a T787A mutation that raised UBE3A activity 517.81% ± 49.3 above WT levels. The individual was diagnosed with failure to thrive, hypotonia, feeding difficulties, and global developmental delay as a child. Autistic behaviors, intellectual disability, and epilepsy were also observed. In addition, Individual 1 was noted to possess small hands and feet, which are typical characteristics seen in Prader–Willi syndrome patients, but no methylation abnormalities in chromosome 15 were detected. The observed phenotypes were not consistent with AS. Individual 1 also had two siblings: one who was typically developing and did not possess the T787A mutation. The other sibling inherited the T787A mutation (noted here as Individual 2) and displayed autistic behaviors and was non-verbal until the age of 4 (Fig. 7b). It was not known at the time of this report if Individual 2 exhibited seizures. Sequencing analysis confirmed the T787A mutation was maternally inherited, however, we were not able to establish the parent-of-origin or the mutation in the mother.

Individual 5 was an adolescent who possessed an in-frame deletion that resulted in the removal of leucine 726 (L726Δ) and raised UBE3A activity 180.75% ± 9.9 above WT levels. The individual presented with global developmental delay, microcephaly, and intractable epilepsy requiring vagal nerve stimulation placement. Delayed motor and language milestones were evident in early childhood and the proband was later diagnosed with quadriplegic cerebral palsy and severe intellectual disability. Individual 5 is currently G-tube dependent for feeding, possesses generalized muscle atrophy and spasticity in the lower extremities, pectus carinatum deformity, severe thoracolumbar scoliosis and thoracic kyphosis. Osteoporosis in the femoral head and neck was also evident. Individual 5 was found to be heterozygous for a pathogenic *ADAR1* variant that was inherited from the mother. However, as *ADAR1* mutation causes an autosomal recessive disorder, this variant was not determined to be causative of disease phenotypes^53,54^. Individual 5 had three siblings. One adolescent sibling (Individual 6), possessed the same L726Δ variant and exhibited intellectual disability and ADHD. The cognitive ability of Individual 6 was estimated at 4 years of developmental age and required special resources at school (Fig. 7c). Individual 6 was not able to read or count and had limited speech. The proband did not exhibit seizures and clinical and behavioral phenotypes, as well as facial features, were not consistent with a diagnosis of AS. A second adolescent sibling did not possess the L726Δ mutation and was typically developing. The final sibling was an adolescent maternal half-sibling who inherited the L726Δ mutation (noted here as Individual 7). Individual 7 was diagnosed with ADHD and was noted for possessing learning difficulties. The proband required an individualized education program (IEP) at school, including special classes for math and social studies. No other abnormalities were noted. Sequencing analysis confirmed the L726Δ mutation was maternally inherited, but the parent-of-origin was unknown for the mother.

## Discussion

Historically, the need to infer the impact of mutations strictly from sequence changes has biased the discovery of disease-causing genes toward lesions that produce loss-of-function alleles. Our functional analyses of missense variants in *UBE3A* revealed that ~12% of variants cause a gain of enzyme function. We found that hyperactivating mutations segregate with affected phenotypes in two unrelated families, and consistent with the neuronal imprinting of *UBE3A*, our mouse modeling and patient data both strongly suggest that hyperactivating mutations confer an increased risk for pathogenicity when present on the maternally-inherited allele.

Our functional variant analysis allowed us to identify a group of mutations in the UBE3A exosite^39^, thereby implicating its dysfunction in UBE3A-dependent disorders. In general, the exosite is thought to facilitate ubiquitin chain elongation by binding the distal ubiquitin on a growing chain and stabilizing ubiquitinated target proteins to the E3 enzyme^41–43,46^. However, this mechanism appears context-dependent as ubiquitin binding to the exosite can also inhibit chain elongation^40^ and ubiquitin variants that bind the exosite with high affinity can alter enzyme activity in complex ways^36^. We also found that additional HECT domain enzymes possess VUS or pathogenic/likely pathogenic variants within their exosites (Supplementary Fig. 4). Examples include NEDD4-2, which is implicated in periventricular nodular heterotopia^37^, HECW2, which is implicated in a neurodevelopmental disorder with hypotonia, seizures, and absent language^55,56^, and HUWE1, which contains a mutation (Y4106C) at a position that is analogous to Q588 in UBE3A and is associated with an X-linked form of intellectual disability^57^ (Supplementary Fig. 4c). Although the functional impact of these variants awaits further study, our observations suggest that exosite dysfunction is a common mechanism that underlies additional neurodevelopmental disorders.

We found that UBE3A hyperactivity causes a general reduction in steady-state enzyme levels due to enhanced self-targeted degradation both in vitro and in vivo (Supplementary Figs. 2 and  5b), a result that is contrary to *UBE3A* duplication, which leads to increased amounts of protein^58^. These observations paint a complex picture about the pathogenic mechanisms of gain-of-function *UBE3A* variants, and our study does not rule out the possibility that such variants may actually cause a loss-of-function state in vivo by lowering overall UBE3A levels in the cell. However, we note that enhanced self-targeting is an enigmatic process that can reflect both enzyme loss and gain-of-function states for UBE3A as well as for other ubiquitin ligase enzymes. Previous studies with various HECT domain enzymes have shown that gain-of-function mutations cause a reduction in steady-state enzyme levels through enhanced self-targeted degradation^12,37,59^, and AS-linked missense mutations in *UBE3A* known to cause constitutive self-targeted degradation^12^ (T106P, T106K, and I130T), produced a loss-of-function response in our BAR assay (Fig. 1d and Supplementary Data 1). In addition, UBE3A is known to undergo self-targeted degradation after the depletion of its substrates^60^, suggesting that heightened self-targeted degradation may be a predicted outcome in the context of enzyme gain-of-function. Additional studies will be required to distinguish these intriguing possibilities and will provide greater insight into the mechanisms of *UBE3A* gain-of-function variants in disease.

Our work strongly suggests that gain-of-function mutations in *UBE3A* confer an increased risk for neurodevelopmental pathology, however, there were variables in our clinical data that limited our ability to elucidate the extent to which excessive UBE3A activity contributes to disease phenotypes. First, our mouse modeling experiments indicated that gain-of-function of maternal UBE3A is required to alter behavioral phenotypes (Fig. 6 and Supplementary Fig. 5). We were able to establish maternal inheritance for some individuals, however, we were unable to determine the parent-of-origin for Individuals 3, 4, 10, 13–17 (Supplementary Table 2). Second, our study could not resolve the contribution of additional genetic and environmental influences on patient phenotypes. For example, Individuals 8 and 10 both possessed variants in *STXBP1*, a gene linked to pediatric seizures and intellectual disability^61^, and Individual 10 was diagnosed with fetal alcohol syndrome (Supplementary Table 2). Moreover, several patients in our cohort possessed skeletal malformations not typically associated with UBE3A dysfunction, reflecting the presence of additional contributing factors for disease. This was particularly notable for Individuals 5–6, who were siblings who possessed the same L726Δ variant. Individual 5 was more severely affected and presented with quadriplegic cerebral palsy, pectus carinatum deformity, severe thoracolumbar scoliosis, and thoracic kyphosis, which were all phenotypes that were absent in the siblings.

Seizures were a prevalent phenotype observed in our patient cohort, and this observation was consistent with the high degree of penetrance of epilepsy in *UBE3A*-dependent disorders^8–11^. However, we stress that careful consideration must be given when attributing this phenotype to gain-of-function mutations in *UBE3A*. A previous study noted that individuals who possess a microduplication encompassing only maternal *UBE3A* exhibited developmental delay, intellectual disability, and behavioral abnormalities, but none of these individuals presented with seizures^14^. In the current study, we observed that seizures were not always a shared phenotype among siblings with the same *UBE3A* gain-of-function mutation (Fig. 7b). For example, seizures were a prominent phenotype in Individual 5, but they were absent in the siblings who possessed the same maternal L726Δ mutation (Individuals 6 and 7). Although we cannot determine at this time whether excessive UBE3A activity is a contributing factor for seizure susceptibility, our study suggests that UBE3A gain-of-function alone is likely insufficient to produce this phenotype.

Finally, our results have broad implications for the nosology of developmental disorders. The results of our study add to the emerging literature suggesting that functional valence can provide a method to distinguish mechanistic subtypes of monogenic disorders. For example, a recent series of studies found that gain-of-function mutations in the sodium channel gene *SCN2A* result in infantile seizures whereas loss-of-function mutations produce autistic phenotypes without seizures^62–64^. Although additional large-scale functional studies of missense variants remain scarce, there are many examples of copy number variations in well-established disease genes that produce distinct disease phenotypes. These include the Rett syndrome gene *MeCP2*^65^, the Fragile X syndrome gene *FMR1*^66^, and *NSD1*, whose deletion^67^ causes Sotos syndrome and duplication^68^ results in a poorly characterized growth disorder. With the increasing development of genetic therapies, understanding such mechanistic differences will be critical for the design and safe implementation of these approaches.

## Methods

### Molecular biology and antibodies

The nomenclature used in this study was based on the amino acid sequence from human UBE3A isoform I (NCBI accession: NP_001361390.1). Myc epitope tags were placed on the N-terminus of UBE3A by polymerase chain reaction and all UBE3A constructs were cloned into pCIG2 plasmid DNA^69^, using SacI and XmaI sites. This vector is driven by a chicken β-actin promoter with bi-cistronic GFP expression through an internal ribosome entry site (IRES), which allowed us to control for transfection efficiency and loading in our in vitro assays. All constructs were verified by sequencing. Disease-linked UBE3A missense mutations used in this study were identified in previous publications^12,21–30^, and are listed in the ClinVar^1^ and gnomAD databases^2^. Cloning for C21Y, R39H, T106P, T106K, C117R, I130T, A178T, N243K, E269G, L273F, I329T, S349P, R477P, M478I, R482P, T485A, L502P, R506C, E550K, and E550L variants were described previously^12^. For most constructs, variants were introduced by PCR using site-specific primers (Integrated DNA Technologies). A complete list of oligonucleotide sequences used in this study are presented in Supplementary Table 3. The STOPΔ variant causes an extension of the C-terminus of UBE3A^70^. Primers matching this extended sequence were designed, annealed, and ligated using an internal SphI site and XmaI. To generate FLAG-tagged RING1B, we placed the epitope tag on the N-terminus of RING1B by polymerase chain reaction using a plasmid encoding a catalytically inactive human RING1B (RING1B I53S)^71^ and cloned into pCIG2 using SacI and XmaI sites. The vector to express the pGEX GST-TEV-Cys-ubiquitin used in the METRIS assay was acquired as a gift from Dr. Brenda Schulman (Max Planck) and described in a previous study^72^. pGL3-BAR and TK-*Renilla* constructs were described previously^12^. The pCDNA3.1 3× FLAG-tagged ubiquitin was a gift from Dr. Mark Zylka (University of North Carolina) and used in a previous study^12^. For UBE3A protein purification, an N-terminal 6×-His tag was added to each UBE3A missense variant by polymerase chain reaction and cloned into the pET-45b(+) plasmid (EMD Millipore) using SacI and NotI restriction sites. Primary antibodies used were mouse anti-Myc (1:1000, Millipore Sigma, #05-419), mouse anti-UBE3A (1:1000, BD Biosciences, #611416), mouse anti-GFP (Santa Cruz Biotechnology #sc-9996), and mouse anti-FLAG (1:1000, #F3165). All secondary antibodies were obtained from LI-COR Biosciences: donkey anti-rabbit 800CW (926-32213), donkey anti-rabbit 680RD (925-68073), donkey anti-mouse 800CW (926-32212), and donkey anti-mouse 680RD (926-68072); all used at a dilution of 1:10,000.

### Cell culture and transfection

HEK293T cells (ATCC) were maintained in a 5% CO_2_ humidified incubator in DMEM containing 25 mM glucose, 0.4 mM glutamine, and 1 mM Sodium Pyruvate (Gibco #10569044), supplemented with 10% fetal bovine serum (Gibco, #16140071) and 1× antibiotic–antimycotic containing penicillin, streptomycin, and amphotericin B (ThermoFisher #15240062). For biochemical analyses, transfections were performed using FuGENE (Promega) according to the manufacturer’s instructions. HEK293T cells were lysed in 2× sample buffer (Fisher NuPAGE™ LDS Sample Buffer #NP0008) supplemented with 5 mM DTT and 1× EDTA-free Pierce protease inhibitor mini tablets (ThermoFisher #A32955). Lysates were boiled and proteins were resolved by 4–20% SDS-PAGE and transferred to nitrocellulose membranes (Bio-Rad). Membranes were blocked in 1× fish gelatin blocking agent (Biotium #22010) and probed with the appropriate primary antibodies overnight at 4 °C or 2 h at RT. Protein bands were visualized using the Odyssey CLx infrared imaging system and Image Studio v5.2 software (LI-Cor Biosciences) and western blots analyzed using ImageJ v2.1.0/1.53c.

### Luciferase assays

BAR assays were performed in 96-well plates for all experiments. HEK293T cells were plated at a density of 10,000/well. Cells were transiently transfected with 10 ng of pRL-TK-*Renilla*, 30 ng of BAR-pGL3, and 30 ng of the indicated constructs using the FuGENE (Promega) transfection reagent, according to the manufacturer’s instructions. Forty-eight hours after transfection, reporter gene expression was assessed using the Dual-Luciferase reporter assay system (Promega) and measured on a Synergy HTX Multi-Mode Reader (BioTek) using Gen5 software v3.08. Luciferase activity was normalized against *Renilla* activity, and UBE3A missense variant signals were normalized to WT UBE3A.

### Protein expression and purification

For protein expression, pET-45b(+) UBE3A constructs were transformed into chloramphenicol-resistant Rosetta™ BL21DE3 competent *E. coli* cells (Sigma-Aldrich #70954). The next day, a colony was picked and transferred into 1 mL of growth media (10 g/L tryptone, 15 g/L yeast extract, 5 g/L NaCl, and 5 g/L K_2_HPO_4_. Just before inoculation, the following was added: 1 mM MgSO_4_, 10 mL/L trace metals (pre-mixed in deionized water and stored at RT: 0.3 g/L CoSO_4_ * 7 H_2_O, 1.5 g/L MnCl_2_ * 4 H_2_O, 0.22 g/L CuSO_4_ * 5 H_2_O, 0.3 g/L H_3_BO_3_, 0.25 g/L Na_2_MoO_4_ * 2 H_2_O, 0.5 g/L ZnCl_2_, 0.93 g/L Na_2_EDTA * 2 H_2_O, and 6.17 g/L FeCl_3_), 0.5% glycerol, 100 µg/mL ampicillin, and 30 µg/mL chloramphenicol). Cultures were allowed to shake at 225 RPM at 37 °C for 2–5 h. Afterwards, 1 mL of culture was transferred into 50 mL of growth media and incubated overnight at 225 RPM at 25 °C. The next morning, 500 mL of induction media (inoculation media + 0.5% glucose) was inoculated to an OD_600_ of 0.4. The induction culture was shaken at 175 RPM at 37 °C until an O.D. of 0.7 was reached (~2 h). Five hundred microliters of 1 M IPTG was then added to induce UBE3A protein expression and the culture was allowed to shake at 175 RPM at 25 °C for 6 h. Cells were pelleted by centrifugation at 1425×*g* at 4 °C for 15 min. For protein purification, pellets were resuspended in 25 mL lysis buffer (25 mM HEPES pH 7.4, 150 mM NaCl, 10% glycerol, 1× EDTA-free Pierce™ protease inhibitor mini tablets (ThermoFisher #A32955), 10 mM imidazole and lysed by sonication. After centrifugation at 15,000×*g* for 15 min at 4 °C, the resulting lysate was incubated with HisPur™ Ni-NTA Resin beads (ThermoFisher #88221) at 4 °C for 60 min. The beads were collected on elution columns and washed with wash buffer (25 mM HEPES pH 7.4, 150 mM NaCl, 10% glycerol, 20 mM imidazole). UBE3A was eluted with 500 mM imidazole. UBE3A was further purified through size-exclusion chromatography and buffer exchange with 7 K MWCO Zeba™ Spin desalting columns (Thermo Scientific #89889) according to the manufacturer’s instructions (final buffer: 25 mM Tris pH 7.5, 150 mM NaCl, 10% glycerol).

### Ubiquitination assays

In vitro ubiquitination assays were performed using human His6 Ubiquitin-activating enzyme/UBE1 recombinant protein (R&D Systems, #E304050), 10× E3 ligase reaction buffer (R&D Systems, #B-71), UbcH7 recombinant protein (R&D Systems, #E2640100), human FLAG-ubiquitin recombinant protein (R&D Systems, #U12002M), and ATP. (Sigma). Assays were performed as described previously^12^. In brief, a single reaction contained 1x E3 ligase reaction buffer, 0.1 µM UBE1, 0.5 µM UBE2L3 (UbcH7), 12.5 µM FLAG-ubiquitin, 2 µM ATP, and 4 µg of purified UBE3A. The reaction was allowed to proceed at room temperature for the indicated times. Reactions were stopped by adding 2× Laemmli buffer and boiling for 5 min. UBE3A ubiquitination was also assayed in HEK293T cells treated with 30 μM MG-132 for 1 h. The cells were then lysed in RIPA buffer containing 1% SDS and 30 μM MG-132. Cell lysates were boiled for 20 min and clarified by centrifugation at 15,000×*g* for 10 min. The resulting supernatant was diluted 1:10 (v/v) in an immunoprecipitation buffer (20 mM HEPES pH 7.4, 50 mM KCl, 1% Triton X-100). For UBE3A immunoprecipitation, an anti-Myc-conjugated affinity gel (Sigma #A7470) was used at 4 °C for 1 h. The final complex was washed three times with wash buffer (immunoprecipitation buffer containing 125 mM NaCl), resuspended in sample buffer, and subject to SDS-PAGE and immunoblot analysis.

### Protein modeling with Rosetta

To model ubiquitin bound to the exosite of UBE3A, we used the Rosetta Relax application through the Rosetta scripts interface^45,73^. To build a starting structure we aligned UBE3A (PDB ID: 1C4Z)^25^ to NEDD4 in the NEDD4-ubiquitin co-complex (PDB ID: 4BBN)^42^, which has a complete ubiquitin tail. We used this starting structure with the relax application in Rosetta to generate 30,000 models of ubiquitin docked to UBE3A. To calculate the RMSD of the ubiquitin tail, we used ProFit with residues 71–76 of ubiquitin. The same protocol was used with WT UBE3A, and the Q588E and Q588R mutations which were generated using the Rosetta fixbb application^74^.

Sequence alignments of UBE3A from various species were performed using amino acid sequences with the following accession numbers: mouse (NP_001029134.1), frog (NP_001080693.1), fish (NP_001007319.1), and fly (NP_648452.1). Sequence alignments of UBE3A with human NEDD4 subfamily enzymes were performed using amino acid sequences from the following accession numbers: NEDD4 (NP_006145.2), NEDD4-2 (NP_001138439.1), HUWE1 (NP_113584.3), HECW1 (NP_055867.3), HECW2 (NP_001335697.1), SMURF1 (NP_001186776.1), SMURF2 (NP_073576.1), WWP1 (NP_008944.1), WWP2 (NP_001257383.1), and ITCH (NP_001311127.1). PDB IDs for additional crystal structures of HECT domains used in this study are as follows: NEDD4-2 (3JVZ), HUWE1 (3H1D), WWP1 (6J1Y), and ITCH (3TUG).

### METRIS assay to measure UBE3A-Ubiquitin interaction

We used a method amenable for measuring weak and transient protein interactions with minimal protein quantities using rolling magnetic probes (RMP). This experiment measures the translational displacement of magnetic particles, immobilized with ubiquitin, on a surface that contains immobilized UBE3A. Streptavidin-biotin was used to immobilize proteins to their respective material. To attach ubiquitin to the magnetic beads we used a commonly used ubiquitin variant with an N-terminal cysteine. Cysteine biotinylation was carried out using Poly(ethylene glycol) [N-(2-maleimidoethyl)carbamoyl]methyl ether 2-(biotinylamino)ethane (Sigma #757748) (Biotin-maleamide). Ubiquitin was singly biotinylated and purified over size exclusion chromatography, and dialyzed to remove excess biotin-maleamide.

The streptavidin coated ferromagnetic particles, provided by Spherotech were composed of a core of polystyrene and CrO_2_. Ten liters of the bead slurry (1.0% w/v) was used to coat with biotinylated-ubiquitin at room temperature for at least 2 h. The amount of protein used was 50× the theoretical limit needed to coat each bead to that ensure all of the binding sites on the beads were occupied (1 mg of beads bound 0.16 nmoles of biotin). Biotinylated UBE3A was fixed to avidin-coated glass slides (Arrayit) with a ligand density of 1.1 × 10^10^ ligands per mm^2^. Microfluidic channels were created on this substrate using two pieces of double-sided tape (3 M). The amount of proteins inserted was enough to coat the channel surface 50× the theoretical limit to ensure that all of the sites on the substrate were coated. The substrate and solution were left in a sealed container for 2 h, then washed to remove excess unbound protein.

Coated ferromagnetic beads were diluted approximately 2000-fold, to reduce the probability of bead aggregation. Beads were placed into channels on the coated slides and sealed. The sealed slide containing the magnetic particles and functionalized substrate was placed in a Helmholtz-inspired apparatus. A clockwise field was actuated at 1 Hz for 5 s, turned off for 5 s, then a counter-clockwise field was actuated at 1 Hz for 5 s, then turned off for 5 s. This actuation cycle was repeated 18 times. Video captured from the CMOS camera was converted into an.avi file using ArcSoft Media Converter 8 then converted into a sequence of jpeg images. Able Particle Tracker was used to track the motion of the beads and the data then imported into Mathematica for analysis.

To analyze the rolling of the particles, we used a dimensionless value called the rolling parameter (ζ) which is a ratio between the maximum possible rolling (i.e., all of the rotational torque is converted to translational displacement) and the observed rolling of the particles. The rolling parameter is given by the following equation:

$$\zeta =\Delta x/\pi D\tau \omega,$$

where *x* is translational displacement, D is the diameter of the beads, τ is the actuation time, and ω is the frequency of the rotation of the magnetic field.

### Fluorescence polarimetry

The HECT domain from WT UBE3A was cloned into pRSFDuet-1 (Novagen) with an Octa-histidine-GST double affinity tag fused to the N-terminus. A TEV protease cleavage sequence was introduced between His-GST and the HECT domain to excise the affinity tag. The UBE3A Q588E mutant was made by site-directed mutagenesis. Both wild type and UBE3A Q588E were transformed into *E. coli* BL21 codon plus RIL (Agilent) cells, and expression was induced by adding 0.6 mM IPTG at 16 °C for 18 h. UBE3A was purified on a nickel affinity gravity column followed by TEV protease digestion and size exclusion purification on an AKTA FPLC (GE healthcare). The purity of the fractions was confirmed by SDS-gel electrophoresis, combined, and concentrated to ~10 mg/mL for biophysical assays. N-terminal fluorescein-labeled ubiquitin (Anaspec) was prepared as described previously^36^. Mixtures of 1 µM fluorescein-labeled ubiquitin and varied concentrations of UBE3A were prepared in a 384-well plate. Milli-fluorescence polarization (mP) values were detected by a Tecan Infinite M1000Pro plate reader at 30 °C. Iterative nonlinear curve fitting using a one-site specific binding model was applied in ProFit to obtain binding affinities (*K*_D_).

### Generation of UBE3A Q588E mice

All animal procedures were performed in accordance with guidelines set by the Washington University Institutional Animal Care and Use Committee. Mice were maintained in standard housing conditions, where food and water were provided ad libitum. Each generation was crossed to wildtype C57BL/J6 mice from Jackson labs.

An analogous Q585E mutation in mouse UBE3A (Q585E, NP_001029134.1) was created by designing single guide RNAs (sgRNAs) to create a C<G substitution at Chr7:59,286,122 (GRCm38/mm10 assembly). The resulting mutation altered a CAG codon into GAG (Fig. S4A). The gRNAs were cloned into the pX330 Cas9 expression plasmid (Addgene), transfected into N2a cells, and validated using the T7 enzyme assay. Validation was performed in the Transgenic Vectors Core at the Washington University School of Medicine. The gRNAs were in vitro transcribed using MEGAShortScript (Ambion) and Cas9 mRNA was in vitro transcribed, G capped and poly-A tailed using the mMessageMachine kit (Ambion). mRNA of Cas9 and the selected gRNA were injected into hybrid C57Bl/6J/JxCBA fertilized eggs at the mouse genetics core at the Washington University School of Medicine. This resulted in 145 founders which were all deep sequenced at the expected cut sites to identify which alleles were present. We identified six founders in which off-target recombination events were not observed, and the Q588E allele represented >90% of the sequenced reads. We selected two founders from this pool and perform additional deep sequencing analysis four kilobases surrounding the targeted region to rule out the presence of additional off-target recombination events.

Founders were crossed to WT C57/Bl6 mice (Jackson Laboratory #000664). Animals were backcrossed for at least six generations prior to use in the study. Mice were genotyped using either tail-tips or toe clippings. Tissue samples were incubated overnight at 55 °C in 400 µL tail lysis buffer (0.1 M Tris pH8.8, 0.2 M NaCl, 0.005 M EDTA, 0.5% SDS + 8 µL proteinase K (Thermo Scientific #BP1700500). Proteinase K was inactivated by incubation at 95 °C for 10 min and genomic DNA purified by isopropanol precipitation. PCR was performed by amplifying a 407 bp fragment with ~200 bp flanking either side of the Q588 codon. The products were purified using a PCR purification kit (Qiagen) and submitted for Sanger sequencing (Genewiz). PCR was performed using GoTaq DNA polymerase (Promega #M3001). An initial denaturation step of 95 °C for 2 min was used followed by 35 cycles of 95 °C for 15 s, 43 °C for 15 s, and an extension step of 72 °C for 30 s.

### Animal behavior

Mice were group housed in standard mouse cages with ad libitum access to food and water, and with a 12 h light/dark cycle in a temperature (~20–22 °C) and humidity-controlled (50% relative humidity) barrier facility. To generate mQ588E animals, heterozygous females possessing the Q588E mutation on the paternal copy of *UBE3A* were crossed with WT C57BL/6 males, and to generate pQ588E animals, heterozygous males possessing the Q588E mutation on the paternal copy were crossed with WT females. For all mQ588E tests used in this paper, a cohort of 45 mice in total was used: 20 female (11 WT, 9 mQ588E) and 25 male (18 WT, 7 mQ588E), and for all pQ588E tests, a cohort of 53 mice was used: 31 female (13 WT and 18 pQ588E) and 21 male (8 WT and 13 pQ588E). Although sex differences were assessed in all experiments, no significant differences observed and data from male and female mice was pooled for our analyses. All behavioral experiments were performed blind to genotype by a female experimenter and also analyzed blind to genotype.

Neonatal motor assessments were performed as described in refs. ^49,50^. Pups were habituated for at least 10 min in the testing room. At P10, grip strength, forelimb suspension, and negative geotaxis was assessed. On P14, grip strength, the surface righting reflex, and the grasping reflex were measured. Each task was performed by each mouse in triplicate and mice were allowed at least 1 min to rest between trials. The values of the three trials were averaged for analysis.

Negative geotaxis was determined by placing the pup head downward on a flat, padded 45° incline and holding it for five seconds to allow it to acclimate. The pup was released, and the time it took the pup to turn to face upward was measured (2 min max). Surface righting reflex was conducted by placing the pup on its back on a flat, padded surface for 5 s and recording the time until the pup positioned itself upright and touched all four paws on the surface. Grasping reflex was determined by holding the mouse vertically by the scruff of the neck, and gently stroking each paw, palm-to-fingertip, five times with the blunt, rounded side of a razor blade. A successful trial was recorded if the pup grasped the blade within five strokes. Forelimb suspension was measured by positioning pups in front of a stable wire strung horizontally across the opening of a wire cup with a padded bottom. Once the mouse grasped the wire with both forepaws, the mouse was released, and the time until the mouse dropped to the padded bottom of the cup was recorded. Grip strength was measured by placing the pup on a plastic mesh screen laid upon a flat, padded surface. Keeping one edge of the screen flat against the table, the screen was slowly rotated (~12 s total) until the screen was inverted (180°). A protractor was placed perpendicularly to the screen to measure the angle at which the pup fell from the screen.

USV recordings were performed as described in ref. ^75^ to assess early communicative behaviors. Pup vocalizations were recorded on postnatal days 5, 7, and 9 in the home colony room of the pups. For each recording session, the dam was placed in a separate cage, and the home cage containing the pups was placed in a warming box (Harvard Apparatus) set to 33 °C to ensure pup temperature in the nest remained between 34–37 °C. An individual pup was selected and its skin temperature taken from the lower dorsal region using a non-contact HDE Infrared Thermometer, as it is known that decreased body temperature results in increased number of USVs^76^. We did not observe a difference in body temperature between genotypes. The pup was then placed in an empty standard mouse cage which in turn was placed within an MDF sound-attenuating box (Med Associates) (36 × 64 × 60 cm). The doors of the sound-attenuating box were shut and USVs were detected using an Avisoft UltraSoundGate CM16 microphone positioned 5 cm above the bottom of the cage, along with the Avisoft UltraSoundGate 116H amplifier and the Avisoft v4.3.01 recorder software. Gain was set to 3 dB and the sampling rate set to 250 kHz. Pups were recorded for 3 min, after which their weight was taken and they were returned to their home cage within the warming box. MATLAB software was used to prepare sonograms of the recordings (frequency range = frequency range = 25–120 kHz, fast Fourier transform size = 512, overlap = 50%, time resolution = 1.024 ms, frequency resolution = 488.2 Hz) using a previously published protocol^75^.

### ClinVar data

The ClinVar database (https://www.ncbi.nlm.nih.gov/clinvar) is a freely accessible, public archive that catalogs relationships among human variants and phenotypes with supporting evidence. Information for this study was collected by searching for missense variants in *UBE3A*. For each variant, the resulting protein change, associated conditions, clinical significance, and accession number was recorded. In addition, de-identified evidence details were recorded if present in the database. For variants without evidence details, the submitting entity was contacted for additional information.

### Statistical analysis

Statistical analysis was performed using GraphPad Prism 7 software and SPSS v27 software for mouse behavior analysis. Statistical treatments for each experiment are listed below:

Figure 1d. One-sample *t*-test (two-tailed) with Benjamini–Hochberg multiple comparisons correction (false discovery rate = 0.05). BAR responses were normalized to WT UBE3A and tested for deviance from a theoretical mean value of 100. Individual *p* values are shown in Table S1. *p < 0.05, **p < 0.005, *p < 0.0005.

Figure 2a. One-sample *t*-test (two-tailed) with Benjamini–Hochberg multiple comparisons correction (false discovery rate = 0.05). *p < 0.05, **p < 0.005, ***p < 0.0005.

Figure 2f. One-sample *t*-test (two-tailed) with Benjamini–Hochberg multiple comparisons correction (false discovery rate = 0.05). *p < 0.05, **p < 0.005, ****p < 0.0005.

Figure 3b, c. One-sample *t*-test (two-tailed) with Benjamini–Hochberg multiple comparisons correction (false discovery rate = 0.05). *p < 0.05, **p < 0.005, ****p < 0.0005.

Figure 5a. One-sample *t*-test (two-tailed) with Benjamini–Hochberg multiple comparisons correction (false discovery rate = 0.05). *p < 0.05, **p < 0.005, ***p < 0.0005.

Figure 5f. Tukey’s multiple comparison test detected a significant interaction between WT UBE3A × Q588E: *q* = 8.580, *p* < 0.001, and between WT UBE3A × Q588R: *q* = 7.004, *p* < 0.001.

Figure 6c, d. Pearson Chi-square test, exact significance (two-sided). *p < 0.05, **p < 0.005, ***p < 0.0005.

Figure 6e, f. Mann–Whitney *U*-test, exact significance (two-tailed). *p < 0.05, **p < 0.005, ***p < 0.0005.

Figure 6h–k. Mann–Whitney *U*-test, exact significance (two-tailed). *p < 0.05, **p < 0.005, ***p < 0.0005.

Figure 6l. Repeated measures ANOVA. *p < 0.05, **p < 0.005, ***p < 0.0005.

Figure 6m. Univariate linear mixed model. *p < 0.05, **p < 0.005, ***p < 0.0005.

Supplementary Fig. 1b. One-sample *t*-test (two-tailed) with Benjamini–Hochberg multiple comparisons correction (false discovery rate = 0.05). *p < 0.05, **p < 0.005, ***p < 0.0005.

Supplementary Fig. 2b. One-sample *t*-test (two-tailed) with Bonferroni multiple comparisons correction (critical vale = 0.05). Mutant enzyme protein levels were normalized to WT UBE3A and tested for deviance from a theoretical mean abundance level of 100. *p < 0.05, **p < 0.005.

Supplementary Fig. 2e. One-sample *t*-test (two-tailed) with Bonferroni multiple comparisons correction (critical value = 0.05). RING1B protein levels for each UBE3A mutant-transfected sample was normalized to WT UBE3A and tested for deviance from a theoretical mean abundance level of 100. *p < 0.05, **p < 0.005.

Supplementary Fig. 3d. One-sample *t*-test (two-tailed) with Benjamini–Hochberg multiple comparisons correction (false discovery rate = 0.05). *p < 0.05, **p < 0.005, ***p < 0.0005.

Supplementary Fig. 5c, f. Mann–Whitney *U*-test, exact significance (two-tailed). *p < 0.05, **p < 0.005, ***p < 0.0005.

Supplementary Fig. 5g–l. Univariate linear mixed model. *p < 0.05, **p < 0.005, ***p < 0.0005.

All behavioral statistical analyses were performed in SPSS (v.27). ANOVA assumption of normality was assessed by using the Shapiro–Wilks test and manual assessment of the *z*-score histogram plot outputs. Mice that had a value greater than 3.29 standard deviations above the mean were deemed influential outliers and were removed from analysis. ANOVA assumption of equal variances was assessed with the Levene’s test. For all reported behavioral tests which were found to violate the assumptions of ANOVA, non-parametric tests were performed. Chi-square goodness of fit tests was used to assess associations between categorical variables. For tests performed across multiple ages, the linear mixed model was used to account for repeated measures.

### Reporting summary

Further information on research design is available in the Nature Research Reporting Summary linked to this article.

## Supplementary information

Supplementary Information

Description of Additional Supplementary Files

Supplementary Data 1

Reporting Summary

## Data Availability

Protein structures used in this study can be found in the Protein Data Bank (PDB) under the following accession numbers, 6U19, 4GIZ1C4Z, 4BBN, 3JVZ, 3H1D, 6J1Y, and 3TUG. Missense variants were identified in the ClinVar database unless otherwise noted, and information was obtained without restrictions on data access. ClinVar accession numbers for variants are listed in Supplementary Data 1. Data supporting the findings in this manuscript are available within the article and in the Source Data file. Source data are provided with this paper.

## Source data

Source Data
