# Supplementary material for: Identification of disease-linked hyperactivating mutations in UBE3A through large-scale functional variant analysis
Source: Nat Commun. 2021 Nov 23;12:6809. doi: 10.1038/s41467-021-27156-0 (PMC8635412; doi:10.1038/s41467-021-27156-0)
Supplement: Supplementary file 1 — Supplementary Information [file 41467_2021_27156_MOESM1_ESM.pdf]

Supplementary Information

Identification of disease-linked hyperactivating mutations in UBE3A through large-scale functional variant analysis

Kellan P. Weston[1‡], Xiaoyi Gao[1‡], Jinghan Zhao[1], Kwang-Soo Kim[1], Susan E. Maloney[2], Jill Gotoff[3], Sumit Parikh[4], Yen-Chen Leu[5], Kuen-Phon Wu[5], Marwan Shinawi[6], Joshua P. Steimel[7], Joseph S. Harrison[8], Jason J. Yi[1,*]

[1]Department of Neuroscience, [2]Department of Psychiatry, [6]Division of Genetics and Genomic Medicine, Department of Pediatrics, St. Louis Children's Hospital, Washington University School of Medicine, St. Louis, MO 63110, USA.

[3]Department of Pediatrics, Geisinger Medical Center, Danville, PA 17822, USA.

[4]Department of Neurogenetics, Neurosciences Institute, Cleveland Clinic, Cleveland, OH 44106, USA.

[5]Institute of Biological Chemistry, Academia Sinica, Taipei, Taiwan.

[7]Deparment of Mechanical Engineering, [8]Department of Chemistry, University of the Pacific, Stockton, CA 95211, USA

[‡]These authors contributed equally to this work

*Correspondence: jasonyi@wustl.edu

Supplementary Note 1

We obtained additional information for two other unrelated individuals who possessed a T787A mutation. Individual 3 was an adolescent found to have intractable epilepsy. Individual 3 was diagnosed with Lennox-Gastaut syndrome without status epilepticus. Individual 4 was an adult diagnosed with intractable migraine accompanied by localization-related epilepsy with complex partial seizures. In both cases, the T787A mutation was identified through a complete epilepsy panel, and we could only obtain limited phenotypic information about these individuals. Importantly, the parental origin of the mutation was not established in either individual. We also identified another mutation at this site (T787M) that raised UBE3A activity 795.38% ± 101.8 above WT levels (Table 1), but we were not able to obtain phenotypic information for this individual.

Individual 8 was a child who possessed a L781H mutation that increased UBE3A activity 615.29% ± 72.7 above WT levels (Supplementary Data 1). The proband had a low APGAR score at birth with good recovery, and no prenatal, pregnancy, or delivery issues. There was no family history of neurodevelopmental disease. Individual 8 was diagnosed initially with developmental delay with hypotonia, feeding issues, and gastroesophageal reflex disease. The individual's functional age was determined to be 15-18 months at the time of examination, and the individual displayed minimal dysmorphism and normal pre and postnatal growth. Epilepsy was observed during infancy and consisted of daily events of behavior arrest, staring, and lip smacking, with occasional eye deviation. Tremulousness in the upper extremities was evident in childhood. Individual 7 exhibited autistic phenotypes including mild sensory issues with light, echolalia, and head banging, but these phenotypes were not consistent with Angelman syndrome. Sequencing confirmed that the L781H mutation was on the maternally-inherited allele. Individual 8 exhibited unusual levels of fatigue and subsequent metabolic studies indicated some mitochondrial dysfunction. Additional sequencing also identified a pathogenic variant in *STXBP1*.

We obtained limited information for Individual 9. The proband possessed an A521T mutation that increased UBE3A activity 464.25% ± 40.0 above WT levels (Supplementary Data 1). The patient exhibited seizures and developmental delay. Sequencing analysis confirmed the mutation was maternally inherited. The mother was unaffected, but had a family history of seizures, suggesting she may have been a carrier for the mutation.

Finally, we identified two additional cases in which the parental origin could not be established. Individual 10 was an adolescent who possessed a R516W mutation that raised activity 812.28% ± 43.8 above WT UBE3A levels. The proband presented with feeding difficulties and began to exhibit staring spells as a child. Individual 10 was diagnosed initially with fetal alcohol syndrome. As a child, the patient's staring spells became frequent and abnormal EEG patterns were evident. The proband failed six different drug treatments to control seizures, and complex seizures including atonic and tonic/clonic seizures were present in childhood. Consistent cognitive delays were also noted without evidence of regression. The proband was not tested for autism due to the extent of the individual's intellectual disability. MRI studies showed that Individual 10 possessed a small right choroidal

tissue cyst and moderate skeletal deformations including mild tibial torsion. Individual 10 was adopted and the parental origin of the *UBE3A* mutation could not be established. Notably, our screen also identified an additional mutation at this site (R516Q) that increased activity 288.95% ± 11.1 above WT enzyme levels. Individual 11 was a child who possessed a G755S mutation that raised UBE3A activity 177.36% ± 12.5 above WT enzyme levels. This individual exhibited seizures and developmental delay. Sequencing analysis determined the mother was negative for the mutation, but it was unknown if the mutation was paternally inherited or *de novo*. Collectively, these data demonstrate that hyperactivating mutations in UBE3A are sufficient to cause a distinct disorder that manifests during early developmental periods.

Supplementary Figure 1

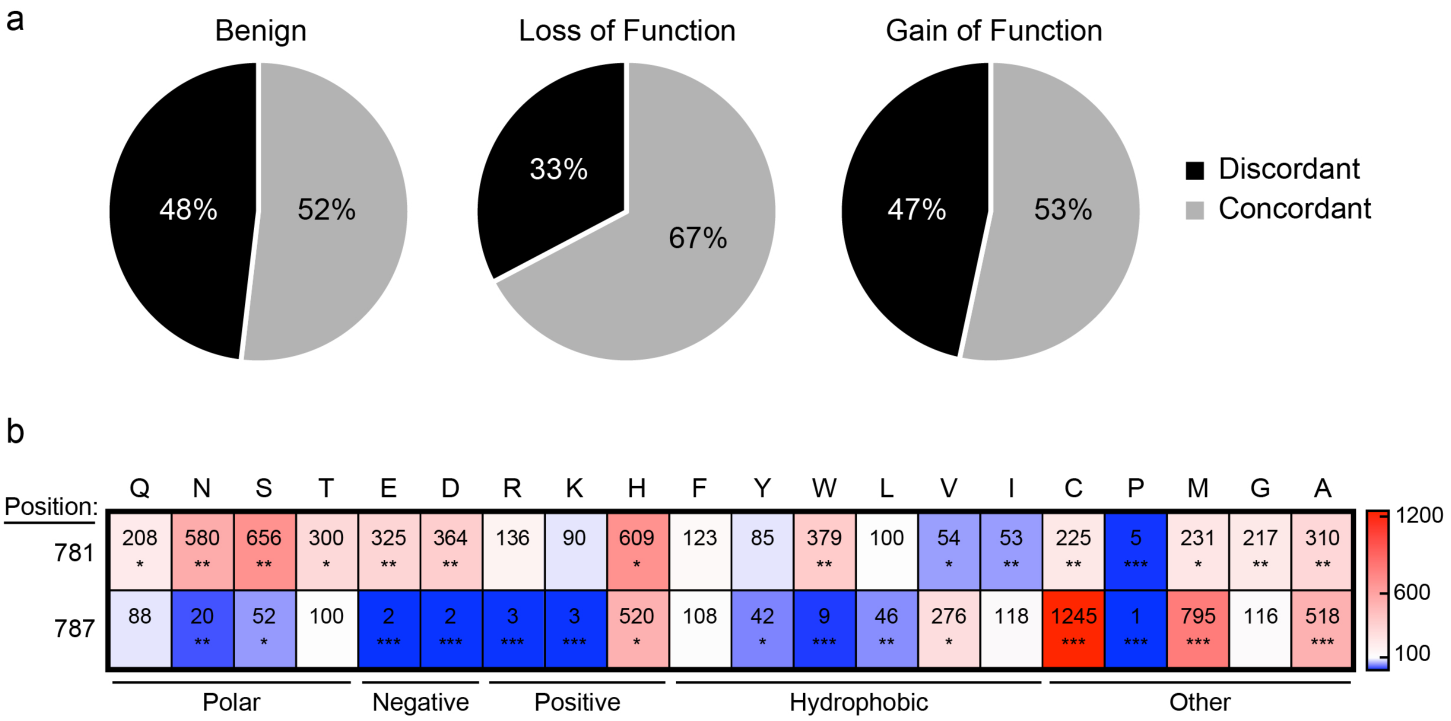

Supplementary Figure 1: Additional characterization of UBE3A variants.

(a) Concordant (gray) and Discordant (black) determinations between functional and *in silico* characterization of *UBE3A* variants tested in this study.

(b) Comprehensive mutational analysis at amino acid positions 781 and 787 in UBE3A. Heat plots show mean responses of mutants in the BAR assay. White shading represents WT UBE3A activity levels, blue shading indicates loss-of-function, and red shading indicates gain-of-function. Scale bar shows the percent change relative to WT UBE3A. *p<0.05, **p<0.005, p<0.0005, One-sample t-test (two-tailed) with Benjamini-Hochberg multiple comparisons correction (FDR = 0.05). Detailed results are provided in Supplementary Table 1.

Supplementary Figure 2

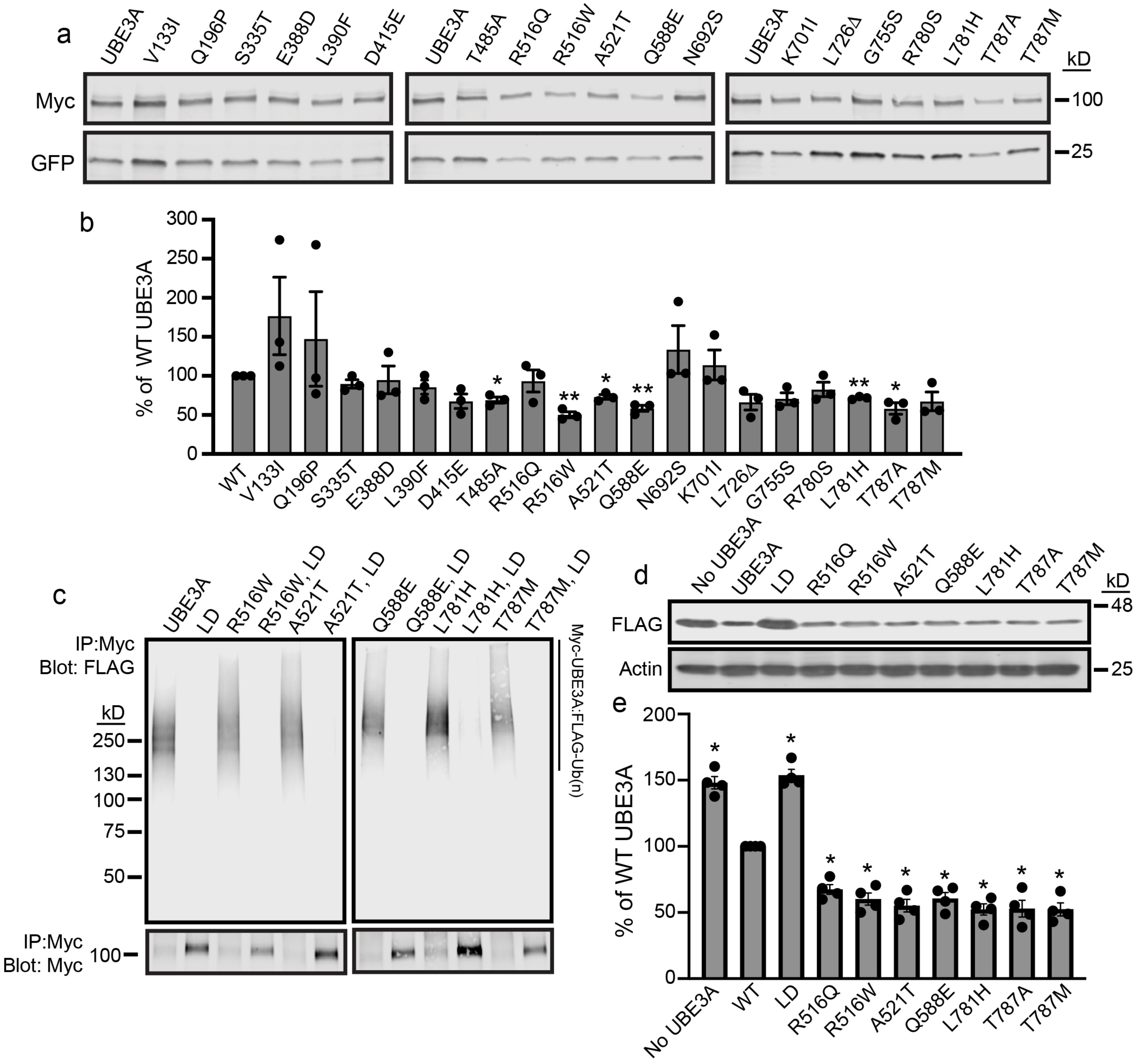

Supplementary Figure 2: Characterization of hyperactive UBE3A mutations.

(a and b) Representative western blot and quantification of HEK293T cells transfected with the indicated constructs. Values are shown as the average percent of WT UBE3A levels ± SE. N = 3 independent experiments, T485A, *p = 0.016; R516W, *p = 0.0063; A521T, p = 0.012; Q588E, *p = 0.009; L781H, *p = 0.0014; T787A, p = 0.03, One-sample t-test (two-tailed) with Bonferroni multiple comparisons correction.

(c) HEK293T cells transfected with the indicated Myc-UBE3A and FLAG-ubiquitin constructs were treated with the proteasome inhibitor MG-132 (30 μM, 1 hr). UBE3A was immunoprecipitated using an anti-Myc antibody and probed by western blot with an anti-FLAG antibody to detect ubiquitinated UBE3A. Representative images are shown from three independent experiments that produced similar results.

(d and e) HEK293T cells were transfected with the indicated Myc-tagged UBE3A constructs and FLAG-tagged RING1B. Protein levels for RING1B were monitored by western blot. Quantification is shown in (e). Values are shown as the mean percent ± SE of WT UBE3A expressing cells, n = 3 independent experiments, No UBE3A, *p = 0.018; UBE3A LD, *p = 0.011; R516Q, *p = 0.027; R516W, *p = 0.029; A521T, *p = 0.022; Q588E, *p = 0.027; L781H, *p = 0.014; T787A, *p = 0.044; T787M, *p = 0.021, One-sample t-test (two-tailed) with Bonferroni multiple comparisons correction..

Supplementary Figure 3

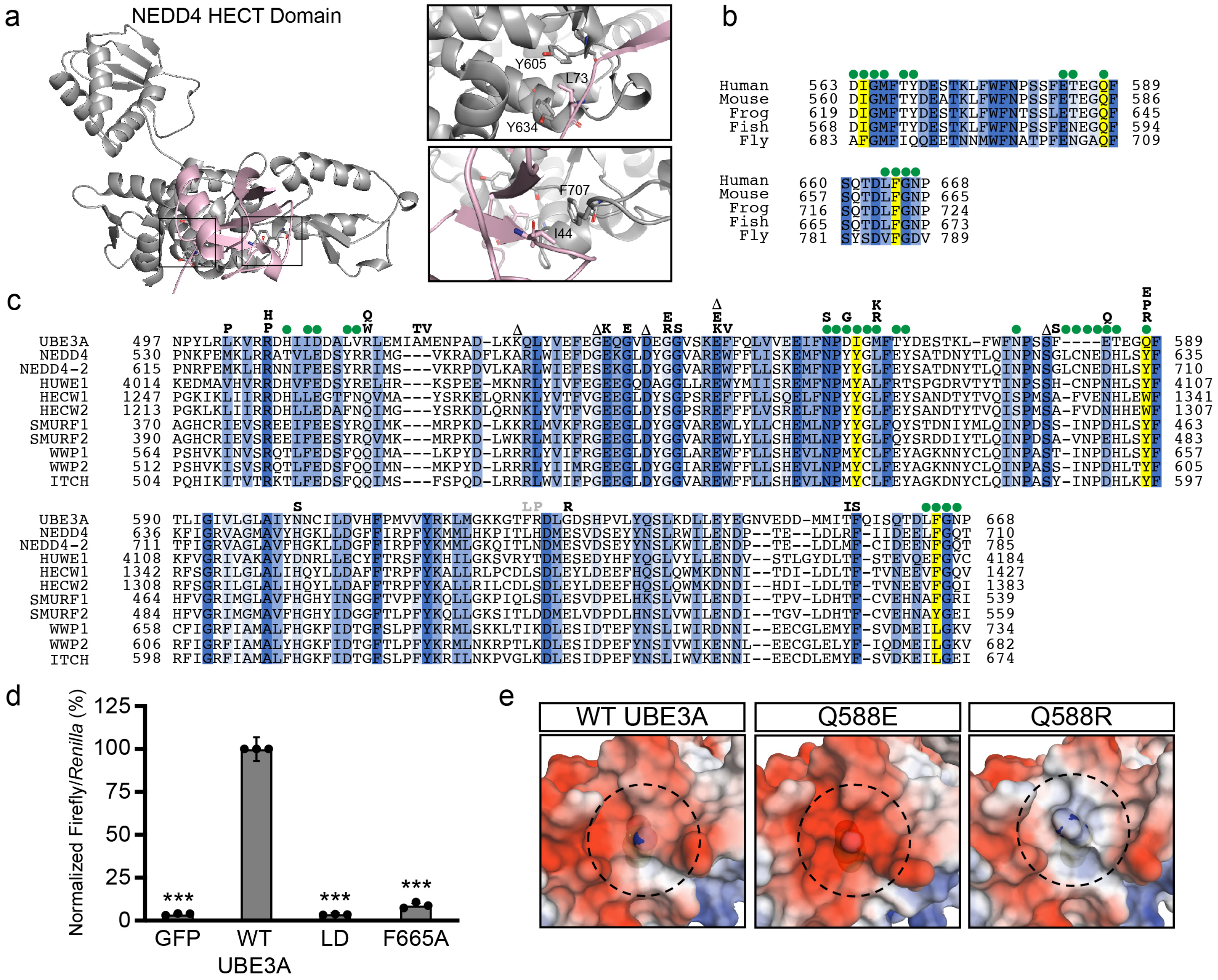

Supplementary Figure 3: UBE3A possesses a degenerate exosite.

(a) The NEDD4 HECT domain (gray) co-crystallized with ubiquitin (pink). Boxed areas show primary contact points consisting of I44 of ubiquitin with F707 of NEDD4, and L73 of ubiquitin with Y605 and Y634 of NEDD4.

(b) Sequence alignment showing the conservation of putative ubiquitin contacting residues (green circles) in UBE3A. Residues highlighted in yellow are critical for the binding of UBE3A to the C-terminus of ubiquitin. Note the high degree of sequence conservation across all species.

(c) Sequence alignment of the exosite of NEDD4 subfamily enzymes with UBE3A. Ubiquitin-contacting residues are marked with green circles. Mutations (black) and benign variants (gray) characterized in this study are shown above the alignment. Residues highlighted in yellow are critical for the binding of NEDD4 sub-family enzymes to the C-terminus of ubiquitin. Note the high degree of conservation of ubiquitin contacting residues in NEDD4 subfamily enzymes and the lack of conservation with UBE3A.

(d) Normalized BAR responses from HEK293T cells transfected with the indicated constructs. Data are shown as the mean $\pm$ SE, n = 3 independent experiments, One-sample t-test with Bonferroni multiple comparisons correction (FDR = 0.05), GFP, ***$p = 1.22 \times 10^{-5}$; UBE3A LD, ***$p = 3.07 \times 10^{-5}$; F665A, ***$p = 1.26 \times 10^{-4}$.

(e) Surface charges at Q588 position are altered by mutations tested in this screen. Positive charges are indicated in blue, negative charges in red, and neutral charged regions in white.

Supplementary Figure 4

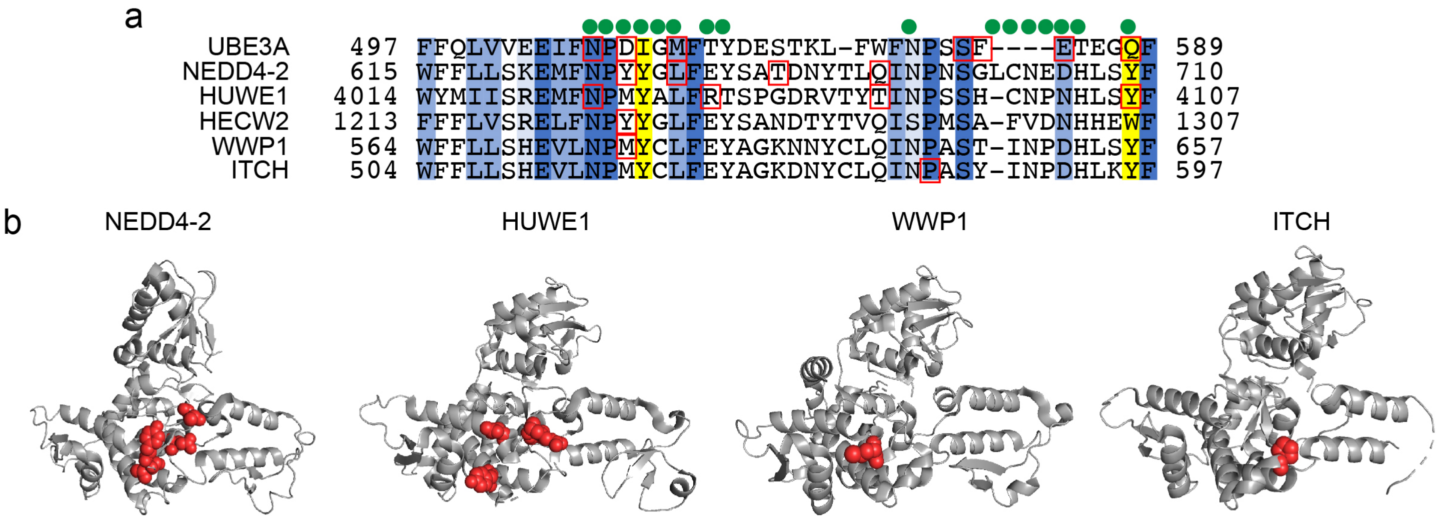

c

Ubiquitin Ligase	Mutation	Clinical Significance	Condition
NEDD4-2	Y679C	Pathogenic	Periventricular nodular heterotopia
	L682F	VUS	Not provided
	T688M	VUS	Not provided
	Q694H	Pathogenic	Periventricular nodular heterotopia
HUWE1	N4075K	Likely Pathogenic	Mental Retardation, X-linked, syndromic, Turner type
	R4082H	VUS	Not specified
	T4092N	VUS	Not provided
	Y4106C	Likely Pathogenic	Mental Retardation, X-linked, syndromic, Turner type
HECW2	Y1277H	Likely Pathogenic	Neurodevelopmental disorder with hypotonia, seizures, and absent language
	R1330W	Pathogenic	Neurodevelopmental disorder with hypotonia, seizures, and absent language
	R1330Q	Likely Pathogenic	Inborn genetic diseases
WWP1	M627L	VUS	Wolf-Parkinson-White pattern
ITCH	P585A	VUS	Autoimmune disease, syndromic multisystem

Supplementary Figure 4: Additional disease-linked exosite mutations in HECT domain enzymes.

(a) Sequence alignment showing amino acids that comprise the exosite. Green circles represent ubiquitin-contact sites. Residues highlighted in yellow are critical for binding to the C-terminus of ubiquitin. Red boxes indicate positions containing disease-linked mutations.

(b) Crystal structures showing the positions of mutations (red) within the exosite of indicated HECT domain enzymes (gray).

(c) List of mutations, and their clinical interpretations in ClinVar with indicated conditions.

Supplementary Figure 5

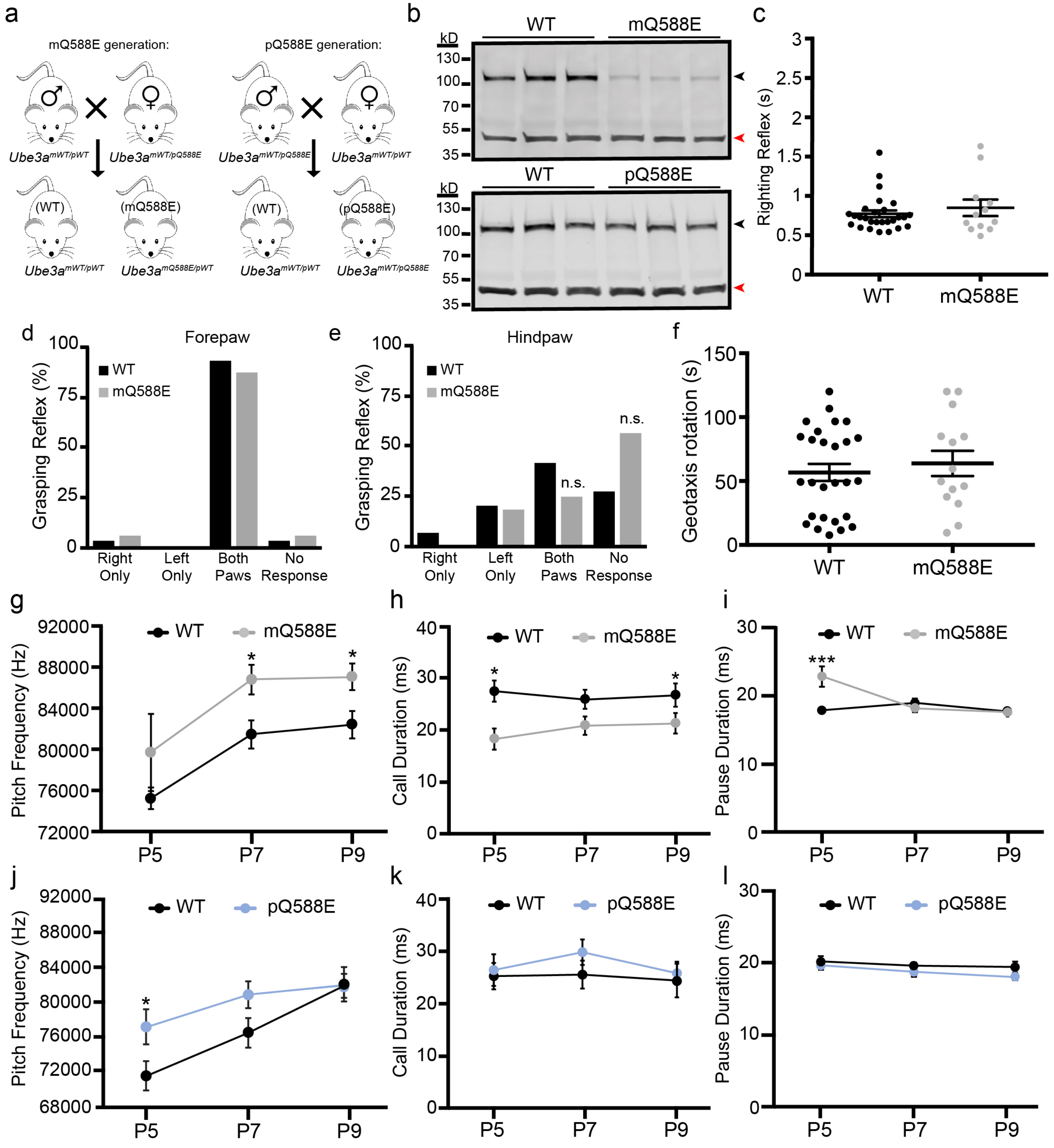

Supplementary Figure 5: The UBE3A Q588E mutation causes neurological deficits in a mouse model.

(a) Mating schemes used to generate heterozygous mice with a maternally-inherited Q588E mutation (mQ588E) and a paternally-inherited Q588E mutation (pQ588E).

(b) Western blot analysis showing UBE3A (black arrowhead) and actin (red arrowhead) protein levels in the cortex from WT, mQ588E, and pQ588E mice. Three mice per genotype were analyzed in each blot.

(c) Individual and mean ± SE for righting times for WT (black) and mQ588E (gray) animals in the righting reflex assay. WT, n = 29 animals; mQ588E, n = 14 animals.

(d and e) Grasping reflexes measured for the forepaw and hindpaw of WT (black) and mQ588E (gray) animals. Values are shown as the percent of tested animals exhibiting the grasping reflex; n.s., not significant. WT, n = 29 animals; mQ588E, n = 16 animals.

(f) Individual and mean ± SE times for WT (black) and mQ588E (gray) mice in the negative geotaxis assay are shown. Each animal was tested three times and the average of the three trials was used for analysis. WT, n = 29 animals; mQ588E, n = 19 animals.

(g) Pitch frequency from ultrasonic vocalization measured between WT (black) and mQ588E (gray) mice. Data are shown as the mean ± SE, *p = 0.01 at P7, *p = 0.048 at P9, Univariate linear mixed model. WT: P5, n = 28 animals, P7, n = 27 animals, P9, n = 24 animals; mQ588E, P5, n = 11 animals, P7, n = 23 animals, P9, n = 17 animals.

(h) Duration of ultrasonic vocalizations measured between WT (black) and mQ588E (gray) mice. Data are shown as the mean ± SE, *p = 0.033 at P5, *p = 0.044 at P9, Univariate linear mixed model. WT: P5, n = 28 animals, P7, n = 27 animals, P9, n = 24 animals; mQ588E, P5, n = 11 animals, P7, n = 20 animals, P9, n = 14 animals.

(i) Pause duration between ultrasonic vocalizations measured between WT (black) and mQ588E (gray) mice. Data are shown as the mean ± SE, ***p = 0.00045, Univariate linear mixed model. WT: P5, n = 28 animals, P7, n = 27 animals, P9, n = 24 animals; mQ588E, P5, n = 11 animals, P7, n = 19 animals, P9, n = 14 animals.

(j) Pitch frequency from ultrasonic vocalization measured between WT (black) and pQ588E (blue) mice. Data are shown as the mean ± SE, *p = 0.033 at P5, Univariate linear mixed model. WT: P5, n = 15 animals, P7, n = 17 animals, P9, n = 13 animals; pQ588E, P5, n = 23 animals, P7, n = 19 animals, P9, n = 22 animals.

(k) Duration of ultrasonic vocalizations measured between WT (black) and pQ588E (blue) mice. Data are shown as the mean ± SE. WT: P5, n = 15 animals, P7, n = 17 animals, P9, n = 13 animals; pQ588E, P5, n = 23 animals, P7, n = 19 animals, P9, n = 22 animals.

(l) Pause duration between ultrasonic vocalizations measured between WT (black) and pQ588E (blue) mice. Data are shown as the mean ± SE. WT: P5, n = 15 animals, P7, n = 17 animals, P9, n = 13 animals; pQ588E, P5, n = 23 animals, P7, n = 19 animals, P9, n = 22 animals.

Supplementary Table 1. Results of comprehensive mutation analysis experiments, related to Figure 2 and Figure 5. Data are shown as the percent change from WT UBE3A responses in the BAR assay. All variants were tested were tested in 3 independent experiments. Luciferase responses were normalized to WT UBE3A and p-values were calculated using a one-sample t-test (two-tailed) with Benjamini-Hochberg multiple comparisons correction (false discovery rate = 0.05).

Amino Acid	Position 588	Standard Error	Adjusted P	Position 781	Standard Error	Adjusted P	Position 787	Standard Error	Adjusted P
Q	100			208.33	13.3424	0.01879	87.97	5.2205	0.1651
N	153.53	6.877	0.306	580.15	26.8470	0.00780	19.98	3.0837	0.0026
S	112.62	6.504	1	656.55	36.4466	0.00833	51.64	5.0179	0.0155
T	125.72	10.744	1	300.34	17.6447	0.01206	100.00	0.0000	
E	389.10	76.462	0.002	335.09	15.6175	0.00833	2.23	0.3203	0.0000
D	320.25	42.140	0.010	363.57	11.1185	0.00674	2.37	0.3693	0.0000
R	5.60	1.917	0.000	136.22	23.4360	0.27685	3.00	0.9161	0.0000
K	6.60	1.621	0.006	89.72	17.0064	0.60711	2.99	0.3570	0.0000
H	12.20	8.907	0.019	308.53	23.9771	0.01759	520.07	49.3264	0.0183
F	10.76	1.551	0.006	123.40	6.6643	0.08600	108.42	8.0221	0.4038
Y	5.10	0.557	0.001	84.56	9.2743	0.26581	41.88	4.8457	0.0109
W	10.42	1.288	0.004	379.07	8.8472	0.00629	8.58	0.4832	0.0001
L	2.70	1.363	0.004	100.00			46.26	1.7135	0.0019
V	19.00	1.223	0.004	53.91	4.2129	0.01206	276.32	26.1131	0.0269
I	25.43	1.549	0.008	52.83	1.7179	0.00629	118.27	8.4108	0.0973
C	36.64	1.972	0.018	224.72	7.1658	0.00780	1245.07	77.9073	0.0001
P	2.19	0.313	0.000	7.49	1.3088	0.00380	1.33	0.2941	0.0000
M	11.09	1.014	0.002	230.89	9.8647	0.00973	795.38	101.7885	0.0002
G	1.72	0.393	0.000	217.41	4.0161	0.00629	116.04	10.6071	0.2846
A	50.05	1.151	0.010	310.25	11.1504	0.00780	517.81	49.3172	0.0000

Supplementary Table 2. Summary of phenotypes from individuals with hyperactivating mutations in UBE3A, related to Figure 7. Phenotypes are summarized for individuals with strong (>150% of WT UBE3A activity) and weak (≤150% of WT UBE3A activity) hyperactivating mutations. *N.D., not determined

Strong hyperactivating mutations (>150% of WT UBE3A activity)

	Individual 1	Individual 2	Individual 3	Individual 4	Individual 5	Individual 6	Individual 7	Individual 8	Individual 9	Individual 10	Individual 11
Mutation (NM_130838.4)	c.2359A>G	c.2359A>G	c.2359A>G	c.2359A>G	c.2176_2178delTTA	c.2176_2178delTTA	c.2176_2178delTTA	c.2402T>A	c.1561G>A	c.1546C>T	c.2263G>A
Protein (NP_001361390.1)	T787A	T787A	T787A	T787A	L726Δ	L726Δ	L726Δ	L781H	A521T	R516W	G755S
Approximate Age at Investigation	Adult	Adolescent	Adolescent	Adult	Adolescent	Adolescent	Adolescent	Child	N.D.	Adolescent	Child
Inheritance	Maternal	Maternal	N.D.	N.D.	Maternal	Maternal	Maternal	Maternal	Maternal	N.D.	Undetermined; mother did not carry mutation, father not tested
Seizures	Yes	N.D.	Yes, intractable epilepsy, diagnosed with Lennox-Gastaut syndrome	Yes, Intractable migraine with localization-related epilepsy	Yes	No	No	Yes, complex seizures, intractable epilepsy	Yes	Yes, complex seizures, intractable epilepsy	Yes
Intellectual Disability	Yes	Yes	N.D.	N.D.	Yes, severe	Yes, cognitive ability equivalent to 4 years of age	Yes, has an individual learning plan in school	Yes, cannot read, cannot perform arithmetic	N.D.	Yes	N.D.
Motor Delays	Yes	N/A	N.D.	N.D.	Yes, quadriplegic cerebral palsy, muscle atrophy	Yes, delayed walking	No	Yes, delayed walking	N.D.	Yes	N.D.
Speech Delays	Yes, Non-verbal	Yes, Non-verbal until age 4	N.D.	N.D.	Yes	Yes, possesses limited speech	No	N.D.	N.D.	Non-verbal	N.D.
Prenatal Feeding Difficulties	Yes	N.D.	N.D.	N.D.	G-tube dependent	N.D.	N.D.	Yes	N.D.	Yes	N.D.
Behavioral Anomalies	ASD	ASD	N.D.	N.D.	N.D.	ADHD, lacks the ability to understand consequences	ADHD	ASD; mild sensory issues with light, echolalia, head-banging	N.D.	ASD	N.D.
Other Anomalies	Failure to thrive, hypotonia, short stature, and global developmental delay noted	N.D.	N.D.	N.D.	Pectus carinatum deformity, severe thoracolumbar scoliosis, thoracic kyphosis	Pyloric stenosis	None	Hypotonia, feeding difficulties, mitochondrial dysfunction, normacytic anemia, GERD	Development delay	Small choroidal cyst, mild tibial torsion	Developmental delay
Other Genetic Anomalies	N.D.	N.D.	N.D.	N.D.	N.D.	Heterozygous for pathogenic mutation in ADAR1 (P193A)	N.D.	Heterozygous WFS1 R456H VUS and heterozygous SPG7 VUS identified; also determined to possess a pathogenic mutation in STXBP1	N.D.	N.D.	N.D.
Additional Notes	Sibling to Individual 2; has an unaffected sibling who does not carry the mutation. Mutation not found in maternal grandmother.	Sibling to Idividual 1; has an unaffected sibling who does not carry the mutation. Mutation not found in maternal grandmother.	N.D.	N.D.	Osteoperosis	Sibling to Individual 5; has an unaffected sibling who does not carry the mutation	Half-sibling to Individual 5	No growth delays observed	Mutation inherited from unaffected mother who has a family history of seizures	Patient was adopted. Was diagnosed with Fetal Alcohol Syndrome	N.D.

Weak hyperactivating mutations (≤150% of WT UBE3A activity)

	Individual 12	Individual 13	Individual 14	Individual 15	Individual 16	Individual 17
Mutation (NM_130838.4)	c.2359A>G	c.2359A>G	c.2359A>G	c.2359A>G		
Protein (NP_001361390.1)	Q196P	Q196P	Q196P	Q196P	N692S	K701I
Approximate Age at Investigation	Child	Child	Adult	Child	Child	Adult
Inheritance	Paternal	N.D.	N.D.	N.D.	N.D.	N.D.
Seizures	No	No	N.D.	Yes, infantile-onset epilepsy	Yes, infantile-onset epilepsy	N.D.
Intellectual Disability	Yes	N.D.	N.D.	N.D.	N.D.	N.D.
Motor Delays	Yes	N.D.	N.D.	N.D.	N.D.	N.D.
Speech Delays	N.D.	N.D.	N.D.	N.D.	N.D.	N.D.
Prenatal Feeding Difficulties	Yes	N.D.	N.D.	N.D.	N.D.	N.D.
Behavioral Anomalies	N.D.	ADHD	N.D.	N.D.	N.D.	N.D.
Other Anomalies	Trigonocephaly, hypotonia, widely spaced teeth, hypoplasia of frontal lobes, global developmental delay, constipation, nasolacrimal duct obstruction, GERD	N.D.	No	Hyperekplexia, severe global developmental delay, sensorineural deafness, decreased vision, hypotonia, spasticity, club feet	N.D.	N.D.
Other Genetic Anomalies	N.D.	Heterozygous VUS in KCTD7 identified	No	Heterozygous VUS in EHMT1, GPHN, and ST3GAL5	Pathogenic variant in STXBP1, VUS in CLN6 and CACNA2D2 identified	N.D.
Additional Notes	N.D.	N.D.	Identified in unaffected female	N.D.	N.D.	Identified in unaffected female

Supplementary Table 3. Primers used in this study. Lower case letters represent codons that were mutated from WT sequence.

Mutation	Sequence
G20V	CAAAACTCATTCGTGCAGGCTTCATTTCCACAgacCTCAGTTAACTGGTGGTAGTAGCGTTCTATTAG
E24K	GACAGGAAGCACAAAACTCATTCGTGCAGGCtttATTTCCACAGCCCTCAGTTAACTGGTGGTAG
R39C	GGCTTTAATAGCTGCTGCATTATTATCCATacaAAGAAAAGTTGGACAGGAAGCACAAAACTC
I47V	GCATTAATCTTATAAAGCTCGAGGGCTTTaacAGCTGCTGCATTATTATCCATACGAAGAAAAG
Y73S	GTTGTTGGGGGCACCTTTCGAGTTCTCAAGggaAGCTGAGCTTGCTCCTTTCTTGGAGGGATG
S77L	CATTTTTATCTCAGAGCAGGAGTTGTTGGGGGCACCTTTcaaGTTCTCAAGGTAAGCTGAGCTTGCTCCTTTCTTGG
M90V	CATCTTTAAAATCAATTCTAGCGCCTTTCTTGTTcacTTTTATCTCAGAGCAGGAGTTGTTGGGGGCACC
R118G	CACGGATTAAAGGGGAATAATCCTCTCTTTCtccACATAATTCAAGAATTTCATATACCTTCTC
R128C	CTCAGCACTAGAAAAAACTCTTCCAATAACacaGATTAAAGGGGAATAATCCTCTCTTTCTCTAC
I130 - V133Δ	GAAGCTCTGTACCAATGCCTCAGCACTAGAAAAAACACGGATTAAAGGGGAATAATCCTCTCTTTC
V133G	CCCCTTTAATCCGTGTTATTGGAAGAggtTTTTCTAGTGCTGAGGCATTGGTACAG
V133I	GCTCTGTACCAATGCCTCAGCACTAGAAAAaatTCTTCCAATAACACGGATTAAAGGGGAATAATC
S135P	CTTTCCGGAAGCTCTGTACCAATGCCTCAGCACTaggAAAAACTCTTCCAATAACACGGATTAAAGGGG
S143R	CTTCCTTGGTGTGTTGTTTAACTTTCCGGAAcctCTGTACCAATGCCTCAGCACTAGAAAAAAC
R145W	CAGTTCTTCCTTGGTGTGTTGTTTAACTTTccaGAAGCTCTGTACCAATGCCTCAGCACTAGAAAA
T151S	CTTTTGCTTGAAGAGATTTCAGTTCTTCCTTgctGTGTTGTTTAACTTTCCGGAAGCTCTGTACC
K170N	CATAGCAGCAGCAGAACATGCAGCTTTTTCattTTCATCTTCATCTTTGTCTTCATCTTTTGC
Q196P	CAGGGCCTAATTTTTGCAAATTGTTGTCTCCcggTGAGCTATCACCTATCCTTGAGGAAGATGC
N200T	CAGACACATCATCAGGGCCTAATTTTTGCAAagtGTTGTCTCCCTGTGAGCTATCACCTATCCTTG
L204S	CATCAATATCCACAGACACATCATCAGGGCCtgaTTTTTGCAAATTGTTGTCTCCCTGTGAGCTATC
D208Δ	CCTTCTAATGGCATCAATATCCACAGACACATCAGGGCCTAATTTTTGCAAATTGTTGTC
D212V	CAATCTGGTGTAGACCCTTCTAATGGCATCAATaacCACAGACACATCATCAGGGCCTAATTTTTGC
S225A	CATTGAGAAAGGCAGTTTCAATTTTTTCATTagcGAGCAATCTGGTGTAGACCCTTCTAATGGC
K228N	CAAATATACAAGTGCATTGAGAAAGGCAGTTTCAATgttTTCATTAGAGAGCAATCTGGTGTAGACCC
L237H	GTCACATTCCACGTTAGGTGACAAATATACatgTGCATTGAGAAAGGCAGTTTCAATTTTTTC
L237P	GTCACATTCCACGTTAGGTGACAAATATACaggTGCATTGAGAAAGGCAGTTTCAATTTTTTC
T249M	TAATTAGGATCTCGAGAGTATACATTGTGATAcatCAAGTCACATTCCACGTTAGGTGACAAATATAC
N252S	CAAATTCAGATAATTAGGATCTCGAGAGTATACactGTGATACGTCAAGTCACATTCCACGTTAGGTG
L263W	GAGATTTCTATTCTCCATTACGATAATGAAccaATTCAGATAATTAGGATCTCGAGAGTATAC
I265T	CTCTCGAGATCCTAATTATCTGAATTTGTTCactATCGTAATGGAGAATAGAAATCTCCACAGTC
V267I	GATCCTAATTATCTGAATTTGTTCATTATCataATGGAGAATAGAAATCTCCACAGTCCTGAATATC
N270T	CATTTCCAGATATTCAGGACTGTGGAGATTTCTagtCTCCATTACGATAATGAACAAATTCAGATAATTAG
L273F	GAATTTGTTCATTATCGTAATGGAGAATAGAAATttcCACAGTCCTGAATATCTGGAAATGGCTTTG
L273R	GCAAAGCCATTTCCAGATATTCAGGACTGTGgcgATTTCTATTCTCCATTACGATAATGAACAAATTC
M281V	GAATAGAAATCTCCACAGTCCTGAATATCTGGAAgtgGCTTTGCCATTATTTTGCAAAGCGATGAGC
A289G	CTGGAAATGGCTTTGCCATTATTTTGCAAAgggATGAGCAAGCTACCCCTTGCAGCCCAAGGAAAAC
A297Δ	GCAAAGCGATGAGCAAGCTACCCCTTGCACAAGGAAAACTGATCAGACTGTGGTCTAAATAC
M316T	GTCTAAATACAATGCAGACCAGATTCGGAGAacgATGGAGACATTTCAGCAACTTATTACTTATAAAG
M317L	GTCTAAATACAATGCAGACCAGATTCGGAGAATGctgGAGACATTTCAGCAACTTATTACTTATAAAG
T325, Y326Δ	GATTTCGACTGTTAAATTCATTGCTTATGACTTTAATAAGTTGCTGAAATGTCTCCATCATTCTC
N331S	CAGCAACTTATTACTTATAAAGTCATAAGCagtGAATTTAACAGTCGAAATCTAGTGAATGATG
S335T	CAATGGCATCATCATCATTCACTAGATTTCGagtGTTAAATTCATTGCTTATGACTTTATAAGTAATAAG
N340Δ	CACTTCGAAGCAGCAACAATGGCATCATCATCCACTAGATTTCGACTGTTAAATTCATTGCTTATG
C351Y	CCACTACATTTGCATAGTAAACCATTTTCAAgtaCTTCGAAGCAGCAACAATGGCATCATCATC
A358G	GCTTCGAAGTGCTTGAAAATGGTTTACTATggaAATGTAGTGGGAGGGGAAGTGGACACAAATC
V360I	GAAGTGCTTGAAAATGGTTTACTATGCAAATataGTGGGAGGGGAAGTGGACACAAATCACAATG
V365M	GGTTTACTATGCAAATGTAGTGGGAGGGGAAatgGACACAAATCACAATGAAGAAGATGATGAAG
N370S	GTAGTGGGAGGGGAAGTGGACACAAATCACagtGAAGAAGATGATGAAGAGCCCATCCCTGAG
E372D	GGAGGGGAAGTGGACACAAATCACAATGAAgatGATGATGAAGAGCCCATCCCTGAGTCCAGCG
D373E	GGGGAAGTGGACACAAATCACAATGAAGAAgaaGATGAAGAGCCCATCCCTGAGTCCAGCGAG
E375D	GTGGACACAAATCACAATGAAGAAGATGATgatGAGCCCATCCCTGAGTCCAGCGAGCTGACAC
I378L	CAAATCACAATGAAGAAGATGATGAAGAGCCCctcCCTGAGTCCAGCGAGCTGACACTTCAGGAAC
P379R	CACAATGAAGAAGATGATGAAGAGCCCATCcgtGAGTCCAGCGAGCTGACACTTCAGGAACTTTTG
E388D	CATCCCTGAGTCCAGCGAGCTGACACTTCAGgacCTTTTGGGAGAAGAAAGAAGAAACAAGAAAG
L390F	GAGTCCAGCGAGCTGACACTTCAGGAACTTttcGGAGAAGAAAGAAGAAACAAGAAAGGTCCTC
P400H	CTTTTGGGAGAAGAAAGAAGAAACAAGAAAGGTcatCGAGTGGACCCCCTGGAAACTGAACTTGGTG
P404R	GAAAGAAGAAACAAGAAAGGTCCTCGAGTGGACcgcCTGGAAACTGAACTTGGTGTTAAAACCCTG
D415E	CTGGAAACTGAACTTGGTGTTAAAACCCTGgaaTGTCGAAAACCACTTATCCCTTTTGAAGAG
L435P	GAAGAGTTTATTAATGAACCACTGAATGAGGTTccaGAAATGGATAAAGATTATACTTTTTTCAAAG
P458L	GAAACAGAGAACAAATTCTCTTTTATGACATGTctcTTTATATTGAATGCTGTCACAAAGAATTTGG
K466E	GACATGTCCCTTTATATTGAATGCTGTCACAgagAATTTGGGATTATATTATGACAATAGAATTC
Y471D	GTACATGCGAATTCTATTGTCATAatcTAATCCCAAATTCTTTGTGACAGCATTC
N474D	ACATGCGAATTCTatcGTCATAATATAATCCCAAATTCTTTGTGAC
M478V	GAATTTGGGATTATATTATGACAATAGAATTCGCgtgTACAGTGAACGAAGAATCACTGTTCTCTACAGC
R506H	GCCGGACAAGTGCATCATCTATGATATGGTCatgTCTAACTTTGAGTCTCAAATATGGATTCAAC
R516Q	CAGGATTTTCCATAGCGATCATCTCTAGctgGACAAGTGCATCATCTATGATATGGTCACG

R516W	CTGCAGGATTTTCCATAGCGATCATCTCTAGccaGACAAGTGCATCATCTATGATATGGTCACG
A521T	CTGCTTCTTCAAGTCTGCAGGATTTTCCATagtGATCATCTCTAGCCGGACAAGTGCATCATC
M522V	CAACTGCTTCTTCAAGTCTGCAGGATTTTCcacAGCGATCATCTCTAGCCGGACAAGTGCATC
K530Δ	CTTGTTCTCCTTCAAATTCCACATACAACTGCTTCAAGTCTGCAGGATTTTCCATAGCGATC
G538Δ	GGAAACACCTCCCTCATCAACTCCTTGTTCTTCAAATTCCACATACAACTGCTTCTTCAAG
E539K	CTTTGGAAACACCTCCCTCATCAACTCCTTGtttTCCTTCAAATTCCACATACAACTGCTTCTTC
G541E	GAAAAAATTCTTTGGAAACACCTCCCTCATCAACttcTTGTTCTCCTTCAAATTCCACATACAACTGC
D543Δ	CAGCTGAAAAAATTCTTTGGAAACACCTCCCTCAACTCCTTGTTCTCCTTCAAATTCCACATAC
G545E	CAACCAGCTGAAAAAATTCTTTGGAAACACCttcCTCATCAACTCCTTGTTCTCCTTCAAATTC
G545R	CAACCAGCTGAAAAAATTCTTTGGAAACACCtctCTCATCAACTCCTTGTTCTCCTTCAAATTCC
G546S	CAACCAGCTGAAAAAATTCTTTGGAAACactTCCCTCATCAACTCCTTGTTCTCCTTC
E550Δ	GAAGATTTCCTCCACAACCAGCTGAAAAAATTTGGAAACACCTCCCTCATCAACTCCTTG
F551V	GGATTGAAGATTTCCTCCACAACCAGCTGAAAaacTTCTTTGGAAACACCTCCCTCATCAACTCCTTG
N561S	GATTCATCGTATGTGAACATACCAATATCTGGactGAAGATTTCCTCCACAACCAGCTGAAAAAATTC
D563G	CAATTTTGTAGATTCATCGTATGTGAACATACCAATaccTGGATTGAAGATTTCCTCCACAACCAGCTG
M566K	CAAAACAATTTTGTAGATTCATCGTATGTGAActtACCAATATCTGGATTGAAGATTTCCTCCAC
M566R	CAAAACAATTTTGTAGATTCATCGTATGTGAAcctACCAATATCTGGATTGAAGATTTCCTCCAC
S582Δ	CCAATCAGAGTAAACTGACCCTCAGTTTCAAAAGATGGATTAAACCAAAACAATTTTGTAGATTC
F583S	CTATGCCAATCAGAGTAAACTGACCCTCAGTTTCagaAGAAGATGGATTAAACCAAAACAATTTTGTAG
E584Q	CTATGCCAATCAGAGTAAACTGACCCTCAGTttgAAAAGAAGATGGATTAAACCAAAACAATTTTG
Q588E	CCAGACCCAGTACTATGCCAATCAGAGTAAActcACCCTCAGTTTCAAAAGAAGATGGATTAAACC
Q588P	CCAGACCCAGTACTATGCCAATCAGAGTAAAcggACCCTCAGTTTCAAAAGAAGATGGATTAAACC
Q588R	GGTTTAATCCATCTTCTTTTGAAACTGAGGGTcggTTTACTCTGATTGGCATAGTACTGGGTCTGG
N602S	CATGGGAAAATGTACATCCAGTATACAGTTactGTAAATAGCCAGACCCAGTACTATGCCAATC
F625L	GATATAGAACTGGGTGAGAGTCTCCCAAGTCACGgaaAGTTCCTTTTTTCCCCATTAGCTTCCTGTAG
R626P	GATATAGAACTGGGTGAGAGTCTCCCAAGTCaggAAAAGTTCCTTTTTTCCCCATTAGCTTCC
G629R	TTTAAACTCTGATATAGAACTGGGTGAGAGTCtcgCAAGTCACGAAAAGTTCCTTTTTTCCCCATTAG
T656I	CCAAAAAGATCTGTCTGTGATATCTGGAAaatGATCATCATGTCATCTTCCACATTCCCTTC
F657S	GTTACCAAAAAGATCTGTCTGTGATATCTGggaAGTGATCATCATGTCATCTTCCACATTCC
L673R	CACTTTCCAGATATCACAGACAGATCTTTTTGGTAACCCAATGATGTATGATcgaAAGGAAAATGGTGATAAAATTCCAATTACAAATG
N676D	GTTTTCATTTGTAATTGGAATTTTATCACCatcTTCCTTTAGATCATACATCATTGGGTTACC
K679T	CAAATTCCTTCCTGTTTTCATTTGTAATTGGAATtgtATCACCATTTTCCTTTAGATCATACATCATTG
F690C	TTATTGAGAATGTAGTCAGAATAAAGATTGACacaTTCCTTCCTGTTTTCATTTGTAATTGGAATTT
V691I	GATTTATTGAGAATGTAGTCAGAATAAAGATTgatAAATTCCTTCCTGTTTTCATTTGTAATTGG
N692H	CTGATTTATTGAGAATGTAGTCAGAATAAAGatgGACAAATTCCTTCCTGTTTTCATTTGTAATTG
N692S	CTGATTTATTGAGAATGTAGTCAGAATAAAGactGACAAATTCCTTCCTGTTTTCATTTGTAATTG
N700DEL	GAATTTGTCAATCTTTATTCTGACTACATTCTCAAATCAGTAGAAAAACAGTTCAAGGCTTTTCG
K701I	GTCAATCTTTATTCTGACTACATTCTCAATataTCAGTAGAAAAACAGTTCAAGGCTTTTCGG
L726Δ	GTGACCAATGAATCTCCCTTAAAGTACTTCAGACCAGAAGAAATTGAATTGCTTATATGTGGAAGC
G738E	CCAGAAGAAATTGAATTGCTTATATGTgaaAGCCGGAATCTAGATTTCCAAGCACTAG
G738R	CAGACCAGAAGAAATTGAATTGCTTATATGTagaAGCCGGAATCTAGATTTCCAAGCACTAGAAG
L747P	GTGGAAGCCGGAATCTAGATTTCCAAGCAccaGAAGAAACTACAGAATATGACGGTGGCTATAC
G755S	CCAAGCACTAGAAGAAACTACAGAATATGACagtGGCTATACCAGGGACTCTGTTCTGATTAGGG
Y757 - D760Δ	CACTAGAAGAAACTACAGAATATGACGGTGGCTCTGTTCTGATTAGGGAGTTCTGGGAAATCG
V762I	GAATATGACGGTGGCTATACCAGGGACTCTattCTGATTAGGGAGTTCTGGGAAATCGTTCATTC
R780S	GAAATCGTTCATTCATTTACAGATGAACAGAAAagtCTCTTCTTGCAGTTTACAACGGGCACAGAC
L781H	GTTCATTCATTTACAGATGAACAGAAAAGAcacTTCTTGCAGTTTACAACGGGCACAGACAGAG
F782Δ	CATTCATTTACAGATGAACAGAAAAGACTCTTGCAGTTTACAACGGGCACAGACAGAGCAC
T787A	GAACAGAAAAGACTCTTCTTGCAGTTTACAgcgGGCACAGACAGAGCACCTGTGGGAGGACTAG
T787M	GAACAGAAAAGACTCTTCTTGCAGTTTACAatgGGCACAGACAGAGCACCTGTGGGAGGACTAG
T787R	GAACAGAAAAGACTCTTCTTGCAGTTTACAaggGGCACAGACAGAGCACCTGTGGGAGGACTAG
K801Δ	CAGAGCACCTGTGGGAGGACTAGGAAAATTAATGATTATAGCCAAAAATGGCCCAGACACAG
M802Δ	GCACCTGTGGGAGGACTAGGAAAATTAAAGATTATAGCCAAAAATGGCCCAGACACAGAAAG
I804DUP	GTGGGAGGACTAGGAAAATTAAAGATGATTataataGCCAAAAATGGCCCAGACACAGAAAGGTTAC
I804K	GTGGGAGGACTAGGAAAATTAAAGATGATTaaaGCCAAAAATGGCCCAGACACAGAAAGGTTAC
G808V	CTAGGAAAATTAAAGATGATTATAGCCAAAAATgtcCCAGACACAGAAAGGTTACCTACATCTCATAC
P815H	GCCAAAAATGGCCCAGACACAGAAAGGTTAcatACATCTCATACTTGCTTTAATGTGCTTTTAC
P815R	GCCAAAAATGGCCCAGACACAGAAAGGTTAcgtACATCTCATACTTGCTTTAATGTGCTTTTAC
C820S	GACACAGAAAGGTTACCTACATCTCATACTagcTTTAATGTGCTTTTACTTCCGGAATACTCAAG
C820Y	GACACAGAAAGGTTACCTACATCTCATACTtacTTTAATGTGCTTTTACTTCCGGAATACTCAAG
P827L	CATCTCATACTTGCTTTAATGTGCTTTTACTTctgGAATACTCAAGCAAAGAAAAACTTAAAGAG
P827S	CATCTCATACTTGCTTTAATGTGCTTTTACTTtcgGAATACTCAAGCAAAGAAAAACTTAAAGAG
Y829H	CATACTTGCTTTAATGTGCTTTTACTTCCGGAAcacTCAAGCAAAGAAAAACTTAAAGAGAGATTG
L835,K836Δ	CTTTTACTTCCGGAATACTCAAGCAAAGAAAAAGAGAGATTGTTGAAGGCCATCACGTATGCC
L835F	CTTTTACTTCCGGAATACTCAAGCAAAGAAAAAtttAAAGAGAGATTGTTGAAGGCCATCACGTATG
G850D	cccgggTTACAGCATgtcAAATCCTTTGGCATACGTGATGGCCTTCAAC
STOPΔ sense	CTGcagaacaagatcaagcagaagaagggcagaaaaaagaaagagaagatcTAAc
STOPΔ antisense	ccgggTTAgatcttctctttcttttttctgcccttcttctgcttgatcttgttctgCAgcatg
Genotyping primer sense	GAATTTTAAGTAACTTAAAGTTAATCAAC
Genotyping primer antisense	CTGAGATAAACTTCTGTATTTAGATAC
