# Supplementary material for: Identification of disease-linked hyperactivating mutations in UBE3A through large-scale functional variant analysis
Source: Nat Commun. 2021 Nov 23;12:6809. doi: 10.1038/s41467-021-27156-0 (PMC8635412; doi:10.1038/s41467-021-27156-0)
Supplement: Supplementary file 2 — Description of Additional Supplementary Files [file 41467_2021_27156_MOESM2_ESM.pdf]

Description of Additional Supplementary Files

File Name: Supplementary Data 1
Description: Summary of variants tested in this study. Data are shown as the percent change from WT UBE3A responses in the BAR assay. All luciferase responses were normalized to WT UBE3A. P-values were calculated using a one-sample t-test (two-tailed) with Benjamini-Hochberg multiple comparisons correction (false discovery rate = 0.05). Published reports of the mutations are indicated when available. All other mutations are catalogued in the ClinVar database.
