# Supplementary material for: Identification of disease-linked hyperactivating mutations in UBE3A through large-scale functional variant analysis
Source: Nat Commun. 2021 Nov 23;12:6809. doi: 10.1038/s41467-021-27156-0 (PMC8635412; doi:10.1038/s41467-021-27156-0)
Supplement: Supplementary file 4 — Reporting Summary [file 41467_2021_27156_MOESM4_ESM.pdf]

Corresponding author(s): Jason J. Yi
Last updated by author(s): 10/29/2021

Reporting Summary

Nature Portfolio wishes to improve the reproducibility of the work that we publish. This form provides structure for consistency and transparency in reporting. For further information on Nature Portfolio policies, see our Editorial Policies and the Editorial Policy Checklist.

Statistics

For all statistical analyses, confirm that the following items are present in the figure legend, table legend, main text, or Methods section.

n/a	Confirmed	
☐	☒	The exact sample size (*n*) for each experimental group/condition, given as a discrete number and unit of measurement
☐	☒	A statement on whether measurements were taken from distinct samples or whether the same sample was measured repeatedly
☐	☒	The statistical test(s) used AND whether they are one- or two-sided<br>*Only common tests should be described solely by name; describe more complex techniques in the Methods section.*
☐	☒	A description of all covariates tested
☐	☒	A description of any assumptions or corrections, such as tests of normality and adjustment for multiple comparisons
☐	☒	A full description of the statistical parameters including central tendency (e.g. means) or other basic estimates (e.g. regression coefficient) AND variation (e.g. standard deviation) or associated estimates of uncertainty (e.g. confidence intervals)
☐	☒	For null hypothesis testing, the test statistic (e.g. *F*, *t*, *r*) with confidence intervals, effect sizes, degrees of freedom and *P* value noted<br>*Give P values as exact values whenever suitable.*
☒	☐	For Bayesian analysis, information on the choice of priors and Markov chain Monte Carlo settings
☒	☐	For hierarchical and complex designs, identification of the appropriate level for tests and full reporting of outcomes
☒	☐	Estimates of effect sizes (e.g. Cohen's *d*, Pearson's *r*), indicating how they were calculated

Our web collection on statistics for biologists contains articles on many of the points above.

Software and code

Policy information about availability of computer code

Data collection	Gen5 software v 3.08 (Biotek) to collect luciferase signals. Li-Cor Image Studio v5.2 for quantitative western blot imaging.
Data analysis	Western blots were analyzed using ImageJ v2.1.0/1.53c. Protein modeling was performed using the Rosetta modeling software v3.12. Binding affinities from fluorescence polarimetry was calculated using ProFit v3.1. Matlab v9.6 was used to generate sonograms from mouse pup ultrasonic vocalization recordings. Statistical analyses were performed using GraphPad Prism 7 software and mouse behavioral data was analyzed using SPSS v27 software.

For manuscripts utilizing custom algorithms or software that are central to the research but not yet described in published literature, software must be made available to editors and reviewers. We strongly encourage code deposition in a community repository (e.g. GitHub). See the Nature Portfolio guidelines for submitting code & software for further information.

Data

Policy information about availability of data

All manuscripts must include a data availability statement. This statement should provide the following information, where applicable:

- Accession codes, unique identifiers, or web links for publicly available datasets
- A description of any restrictions on data availability
- For clinical datasets or third party data, please ensure that the statement adheres to our policy

Protein structures used in this study can be found in the Protein Data Bank (PDB) under the following accession numbers, 6U19 (http://doi.org/10.2210/pdb6U19/pdb), 4GIZ (http://doi.org/10.2210/pdb4GIZ/pdb, 1C4Z (http://doi.org/10.2210/pdb1C4Z/pdb), 4BBN (http://doi.org/10.2210/pdb4BBN/pdb), 3JVZ (http://doi.org/10.2210/pdb3JVZ/pdb), 3H1D (http://doi.org/10.2210/pdb3H1D/pdb), 6J1Y (http://doi.org/10.2210/pdb6J1Y/pdb), 3TUG (http://doi.org/10.2210/

pdb3TUG/pdb). Missense variants were identified in the ClinVar database unless otherwise noted. ClinVar accession numbers for variants are listed in Supplementary Table 1. Data supporting the findings in this manuscript are available within the article and in the Source Data file.

Field-specific reporting

Please select the one below that is the best fit for your research. If you are not sure, read the appropriate sections before making your selection.

☒ Life sciences ☐ Behavioural & social sciences ☐ Ecological, evolutionary & environmental sciences

For a reference copy of the document with all sections, see nature.com/documents/nr-reporting-summary-flat.pdf

Life sciences study design

All studies must disclose on these points even when the disclosure is negative.

Sample size	Sample sizes were chosen based on our experience and publications using similar methodology [Yi et al. J Biol Chem (2017), 292(30):12503-12515, doi: 10.1074/jbc.M117.788448; Kuhnle et al., J Biol Chem (2018), 293(47):18387-18399, doi: 10.1074/jbc.RA118.004653]. To assess motor behaviors in ice, we performed power calculations using G*Power v 3.1 software using estimated effect sizes previously observed for genotype in Ube3a-null animals (f=0.68; Born et al. Sci Rep (2017), 7(1):8451, doi: 10.1038/s41598-017-08825-x). Our calculations indicated a total sample size of 14 animals per group would be needed to detect a similar effect of genotype with 80% power using ANOVA with alpha at 0.05. Similarly, power calculations for ultrasonic vocalizations indicated that a sample size of 22 animals per group would be needed to detect a large effect (f=0.5) of genotype with 80% power using ANOVA with alpha at 0.05. There is no evidence of a sex effect in UBE3A-dependent disorders, so this was not a focus of our analyses.
Data exclusions	Data was only excluded from behavioral analyses by assessing ANOVA assumption of normality using the Shapiro-Wilks test and manual assessment of the z-score histogram plot outputs. Mice that had a value greater than 3.29 standard deviations above the mean were deemed influential outliers and were removed from analysis.
Replication	All in vitro experiments were replicated at least three times. This includes the experiments in Fig. 3d and Fig. S2C, and all attempts yielded successful results.
Randomization	The allocation of all samples in our study was random and animals were tested in randomized cohorts for behavioral analysis.
Blinding	All behavioral experiments were performed blind to genotype by a female experimenter and also analyzed blind to genotype. Experimenters were blind to genotypes for data collection as well as during data analysis.

Reporting for specific materials, systems and methods

We require information from authors about some types of materials, experimental systems and methods used in many studies. Here, indicate whether each material, system or method listed is relevant to your study. If you are not sure if a list item applies to your research, read the appropriate section before selecting a response.

Materials & experimental systems

n/a	Involved in the study	
☐	☒	Antibodies
☐	☒	Eukaryotic cell lines
☒	☐	Palaeontology and archaeology
☐	☒	Animals and other organisms
☒	☐	Human research participants
☒	☐	Clinical data
☒	☐	Dual use research of concern

Methods

n/a	Involved in the study	
☒	☐	ChIP-seq
☒	☐	Flow cytometry
☒	☐	MRI-based neuroimaging

Antibodies

Antibodies used	anti-Myc (1:1000, Millipore Sigma, #05-419), mouse anti-UBE3A (1:1000, BD Biosciences, #611416), mouse anti-GFP (Santa Cruz Biotechnology #sc-9996), and mouse anti-FLAG (1:1000, #F3165). From LI-COR Biosciences: donkey anti-rabbit 800CW (926-32213), donkey anti-rabbit 680RD (925-68073), donkey anti-mouse 800CW (926-32212), and donkey anti-mouse 680RD (926-68072); all used at a dilution of 1:10,000.
Validation	All antibodies used were selected after searching a publicly available database that tracks reagent usage in the literature (www.citeab.com) and further validated in our laboratory. To validate Myc, GFP, and FLAG antibodies, we performed western blot analysis using cell lysates from untransfected HEK293T cells. UBE3A antibody validation was performed using brain lysates from mice lacking a copy of maternal UBE3A, which causes the loss of UBE3A expression in neurons. We do not include this information in the current manuscript. Secondary antibodies used in the study were all purchased from LI-COR Biosciences and were as follows: donkey anti-rabbit 800CW (926-32213), donkey anti-rabbit 680RD (925-68073), donkey anti-mouse 800CW (926-32212), and donkey anti-mouse 680RD (926-68072).

Eukaryotic cell lines

Policy information about cell lines

Cell line source(s)	HEK293T (ATCC)
Authentication	HEK293T cells were used directly from the supplier. Separate validation was not performed in our laboratory.
Mycoplasma contamination	Cells were not tested for mycoplasma contamination.
Commonly misidentified lines (See ICLAC register)	No commonly misidentified cell lines were used in this study.

Animals and other organisms

Policy information about studies involving animals; ARRIVE guidelines recommended for reporting animal research

Laboratory animals	C57/bl6j Mice, age postnatal day 5 (P5) - P30
Wild animals	No wild animals were used in this study.
Field-collected samples	No field-collected samples were used in this study.
Ethics oversight	This study was approved by the Institutional Animal Care and Use Committee at the Washington University School of Medicine.

Note that full information on the approval of the study protocol must also be provided in the manuscript.
